# Supplementary figures and images for: Leveraging auxiliary data from arbitrary distributions to boost GWAS discovery with Flexible cFDR
Source: PLoS Genet. 2021 Oct 20;17(10):e1009853. doi: 10.1371/journal.pgen.1009853 (PMC8559959; doi:10.1371/journal.pgen.1009853)

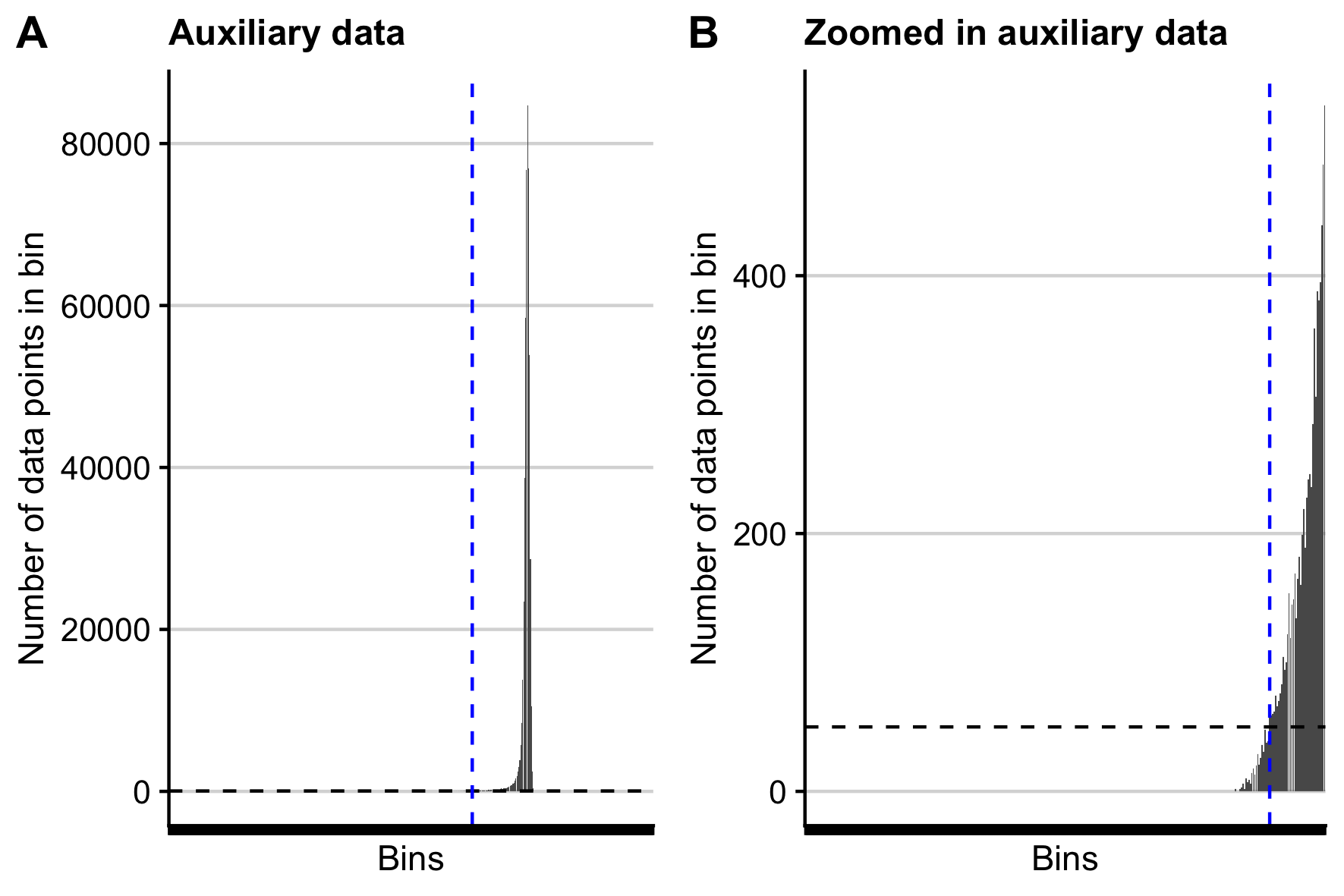

Supplement: S1 Fig — Plots showing how many data points are in each grid space of the auxiliary data, q, over the support of the KDE for an example data set. (A) shows the full support of the KDE and (B) is zoomed in to the left tail. Black dashed line at y = 50 which is the default value of the gridp parameter in the fcfdr::flexible_cfdr function. Data points falling in grid spaces with fewer than 50 data points (those to the left of the blue dashed line) are left-censored, meaning that their value is replaced by the value of the left bound of the first grid space containing more than 50 data points. In practise, very few data points are left-censored. (TIF) [file pgen.1009853.s001.tif]

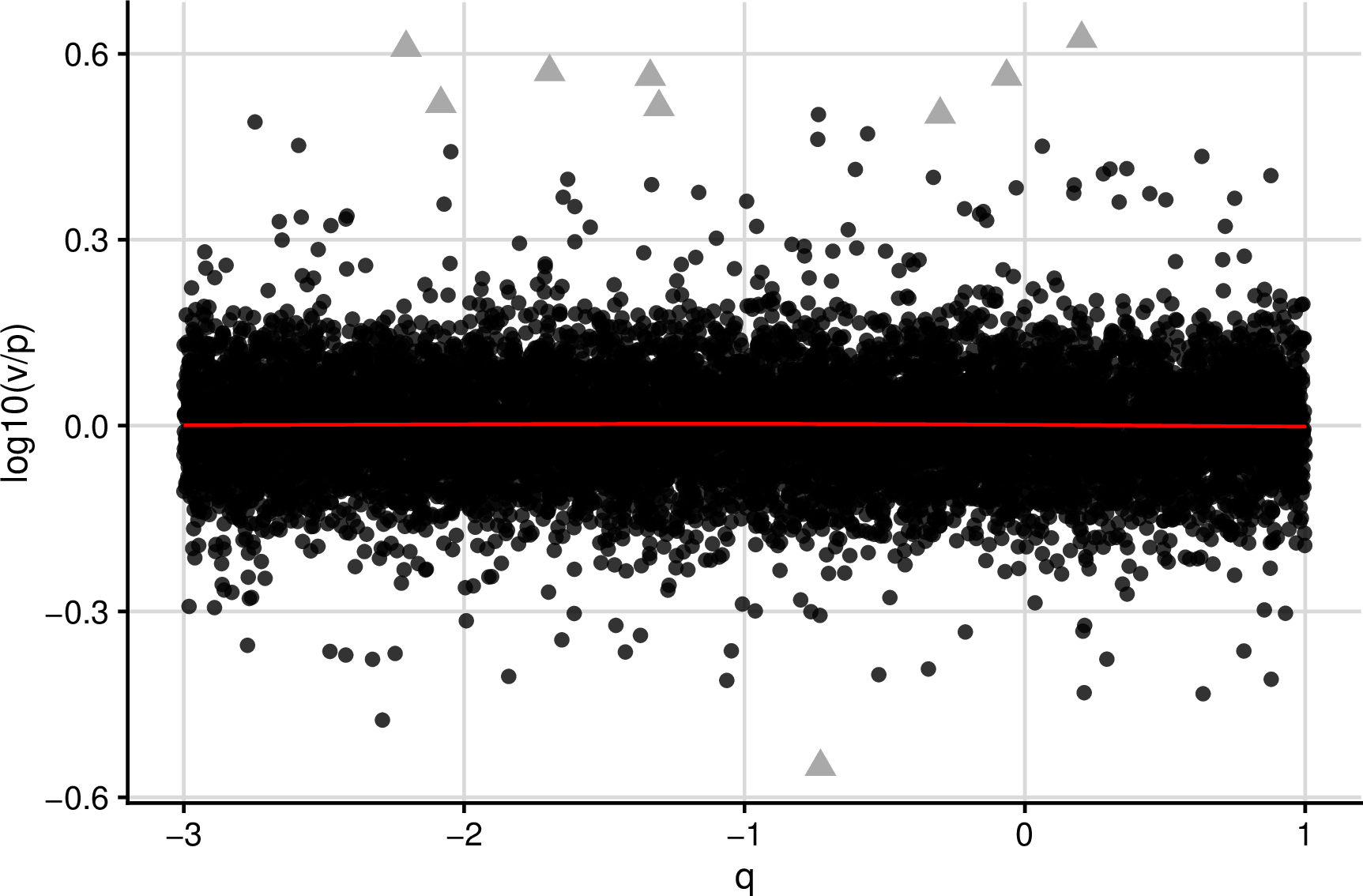

Supplement: S2 Fig — A spline with 5 knots is fitted to log10(v/p) against q using the bigsplines R package (https://cran.r-project.org/web/packages/bigsplines/index.html) for an example data set. The distance between each data point and the fitted spline is calculated. If this distance is greater than the value of the dist_thr parameter in the fcfdr::flexible_cfdr function (default value is 0.5), then the data point is mapped back to the spline and the corresponding v-value is recalculated using the fitted spline. In this example, the red line shows the fitted spline and the grey triangular points are mapped back to the spline to generate new v-values. (TIF) [file pgen.1009853.s002.tif]

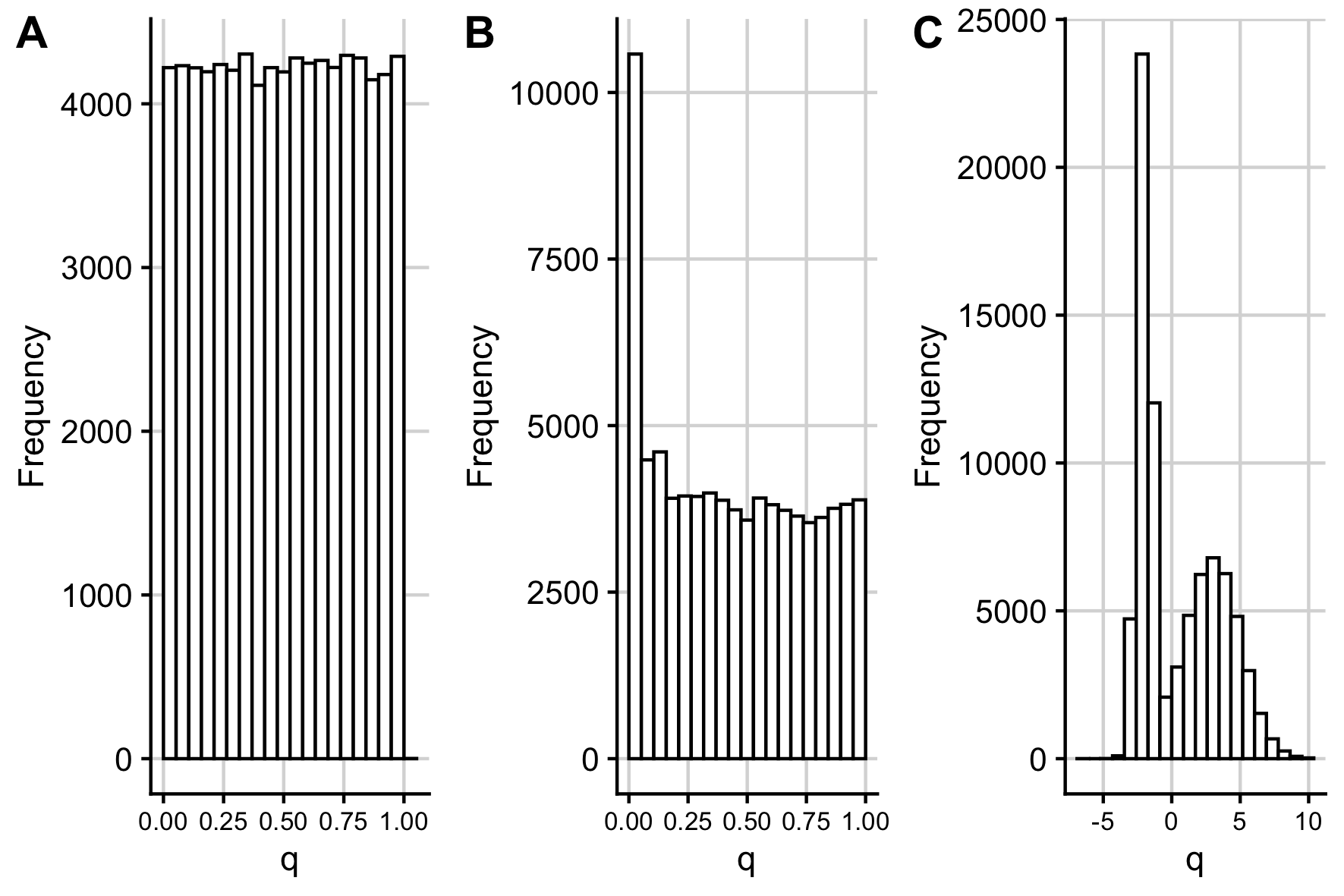

Supplement: S3 Fig — (A) Example data leveraged in simulation A (simulated from standard uniform distribution). (B) Example data leveraged in simulation B (simulated p-values for related traits). (C) Example data leveraged in simulations C, D and E (simulated from a mixture normal distribution). (TIF) [file pgen.1009853.s003.tif]

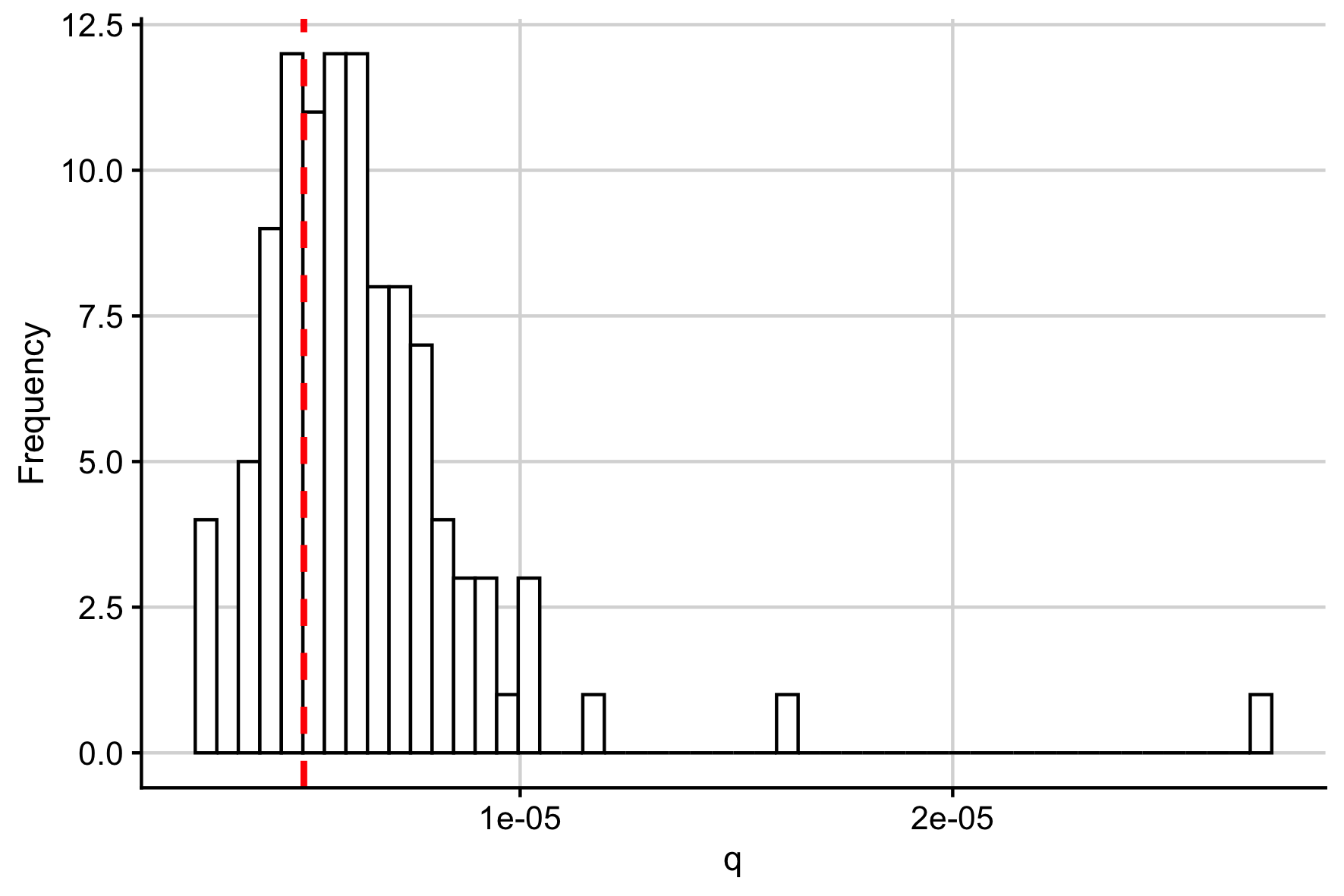

Supplement: S4 Fig — Histogram of the maximum FDR-adjusted p-value (using BH method) amongst SNPs with p ≤ 5 × 10^−8^ in the simulation analysis. Red dashed line at the selected FDR threshold of 5 × 10^−6^. (TIF) [file pgen.1009853.s004.tif]

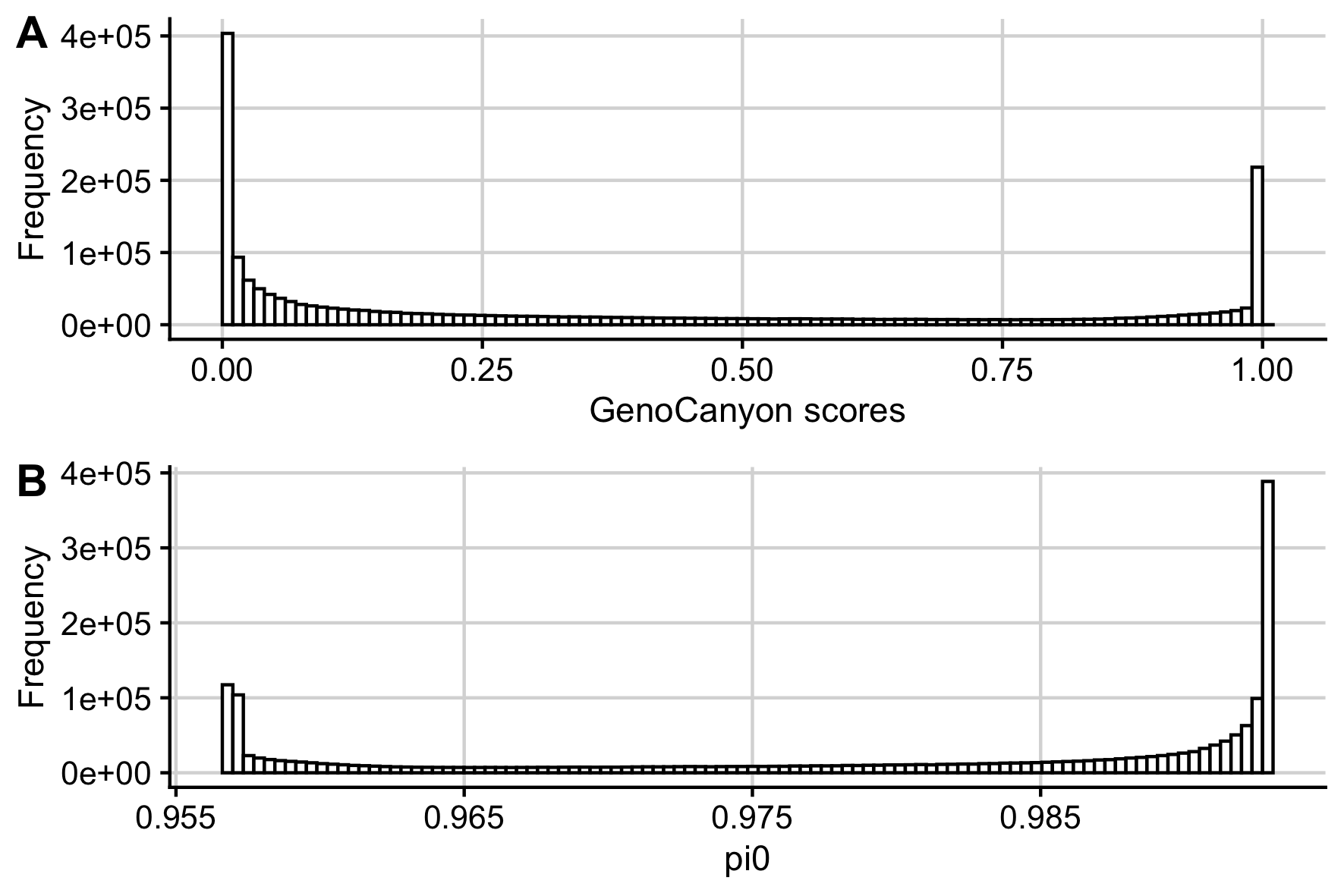

Supplement: S5 Fig — (A) Histogram of GenoCanyon scores for SNPs in the asthma GWAS data set. (B) Histogram of estimated pi0 values from BL. (TIF) [file pgen.1009853.s005.tif]

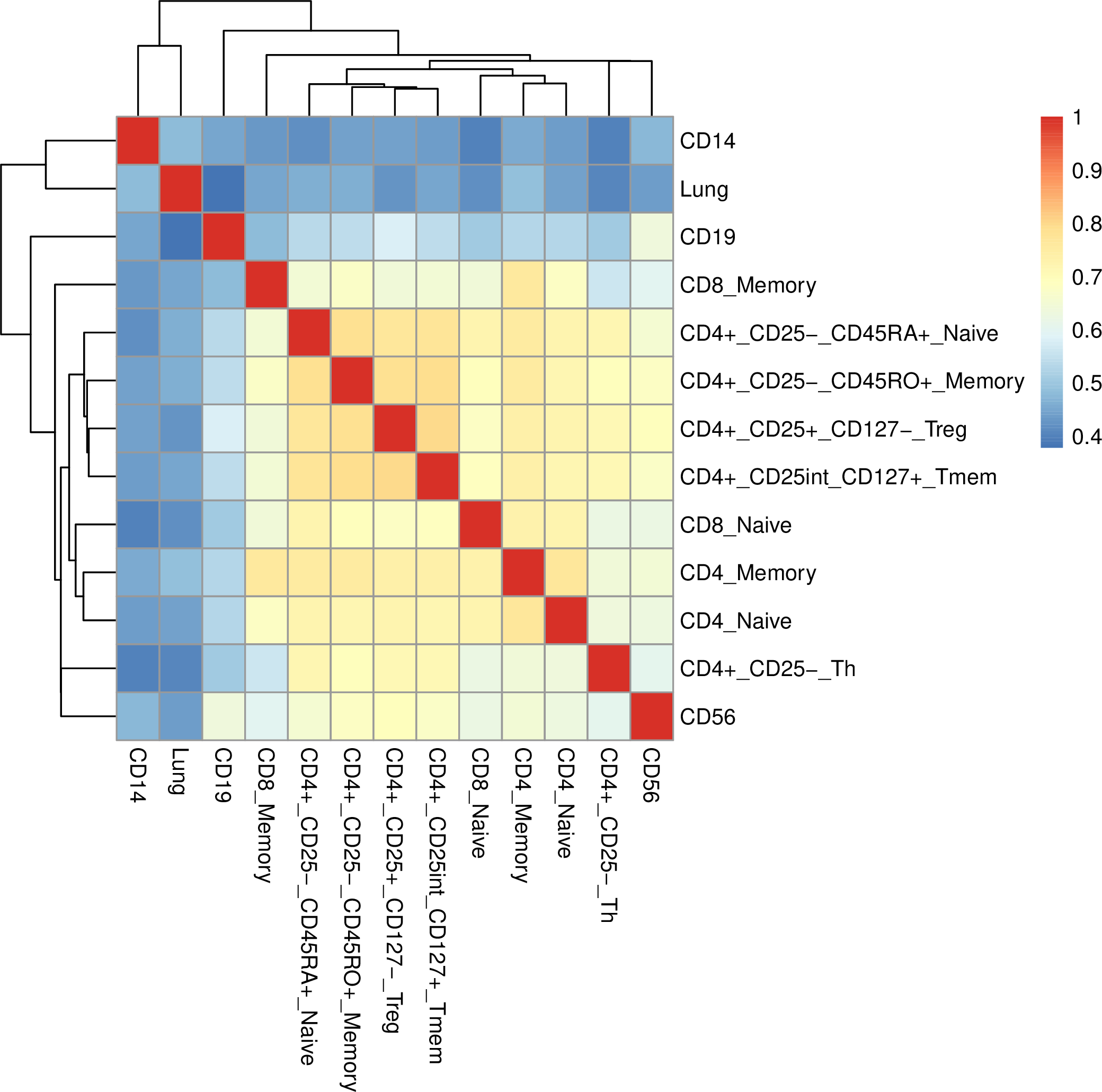

Supplement: S6 Fig — (TIF) [file pgen.1009853.s006.tif]

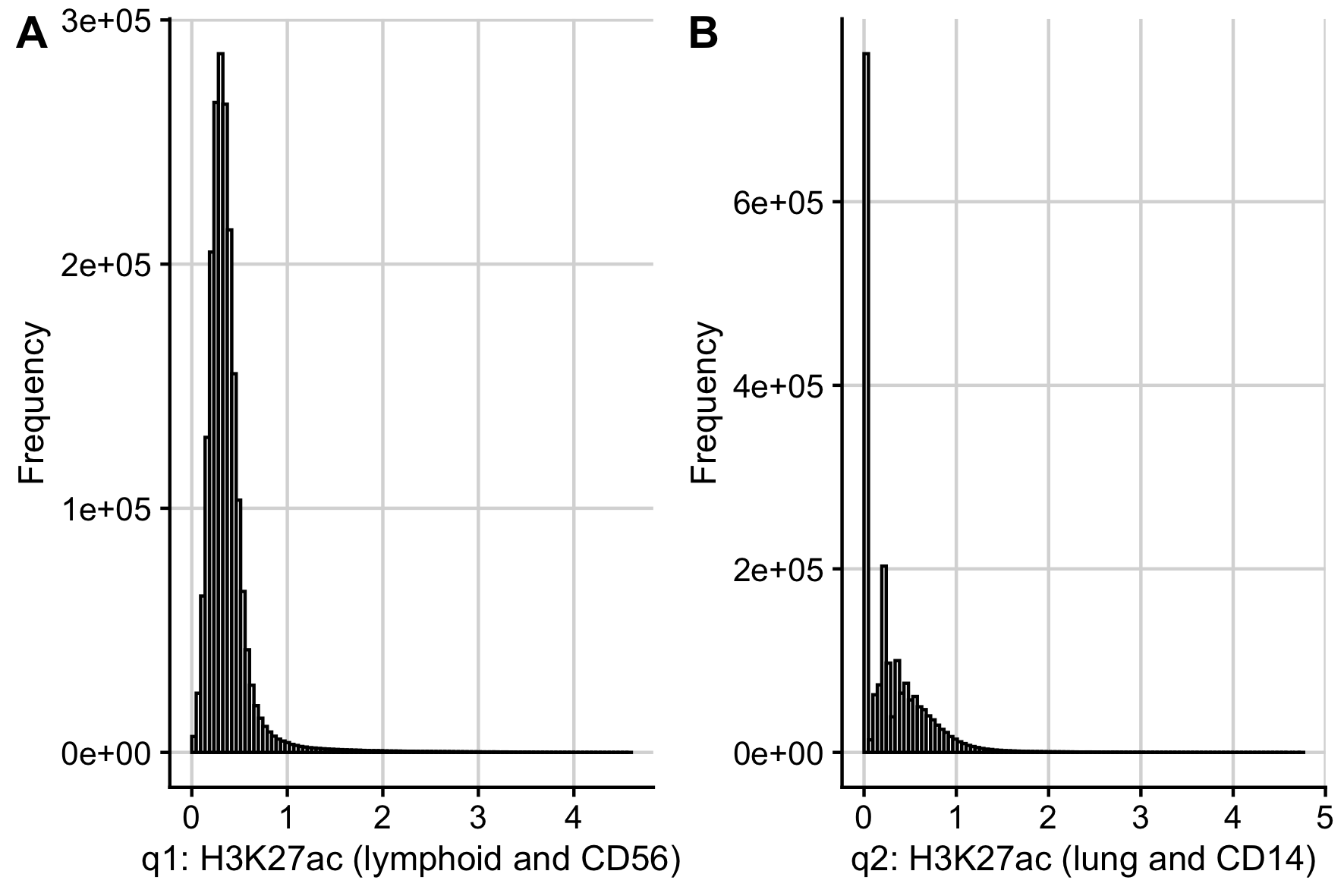

Supplement: S7 Fig — (A) q1 is the average of (log transformed) H3K27ac fold change values in lymphoid and CD56 cell types (B) q2 is the average of (log transformed) H3K27ac fold change values in lung tissue and CD14+ cells. (TIF) [file pgen.1009853.s007.tif]

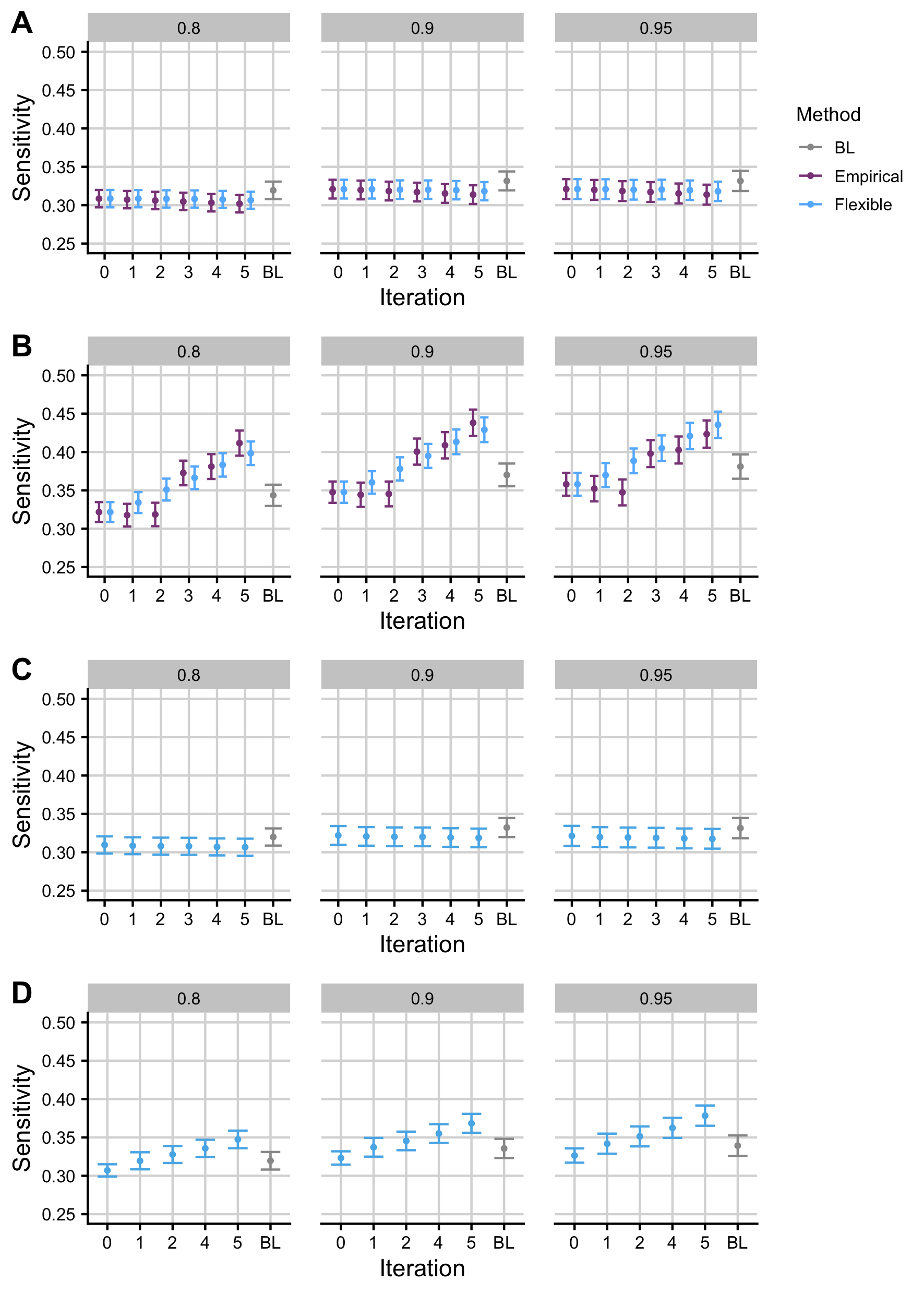

Supplement: S8 Fig — Mean +/- standard error for the sensitivity of FDR values from empirical and Flexible cFDR when iterating over independent (A; “simulation A”) and dependent (B; “simulation B”) auxiliary data that is bounded by [0, 1]. Panels C and D show the results from Flexible cFDR when iterating over independent (C; “simulation C”) and dependent (D; “simulation D”) auxiliary data simulated from bimodal mixture normal distributions. BL refers to results when using Boca and Leek’s FDR regression to leverage the 5-dimensional covariate data. Iteration 0 corresponds to the original FDR values. Our sensitivity proxy is calculated as the proportion of SNPs with r2 ≥ X with a causal variant (“truly associated”), that were detected with a FDR value less than 5 × 10^−6^, where results are faceted for X = 0.8, 0.9, 0.95. Results were averaged across 100 simulations. (TIF) [file pgen.1009853.s008.tif]

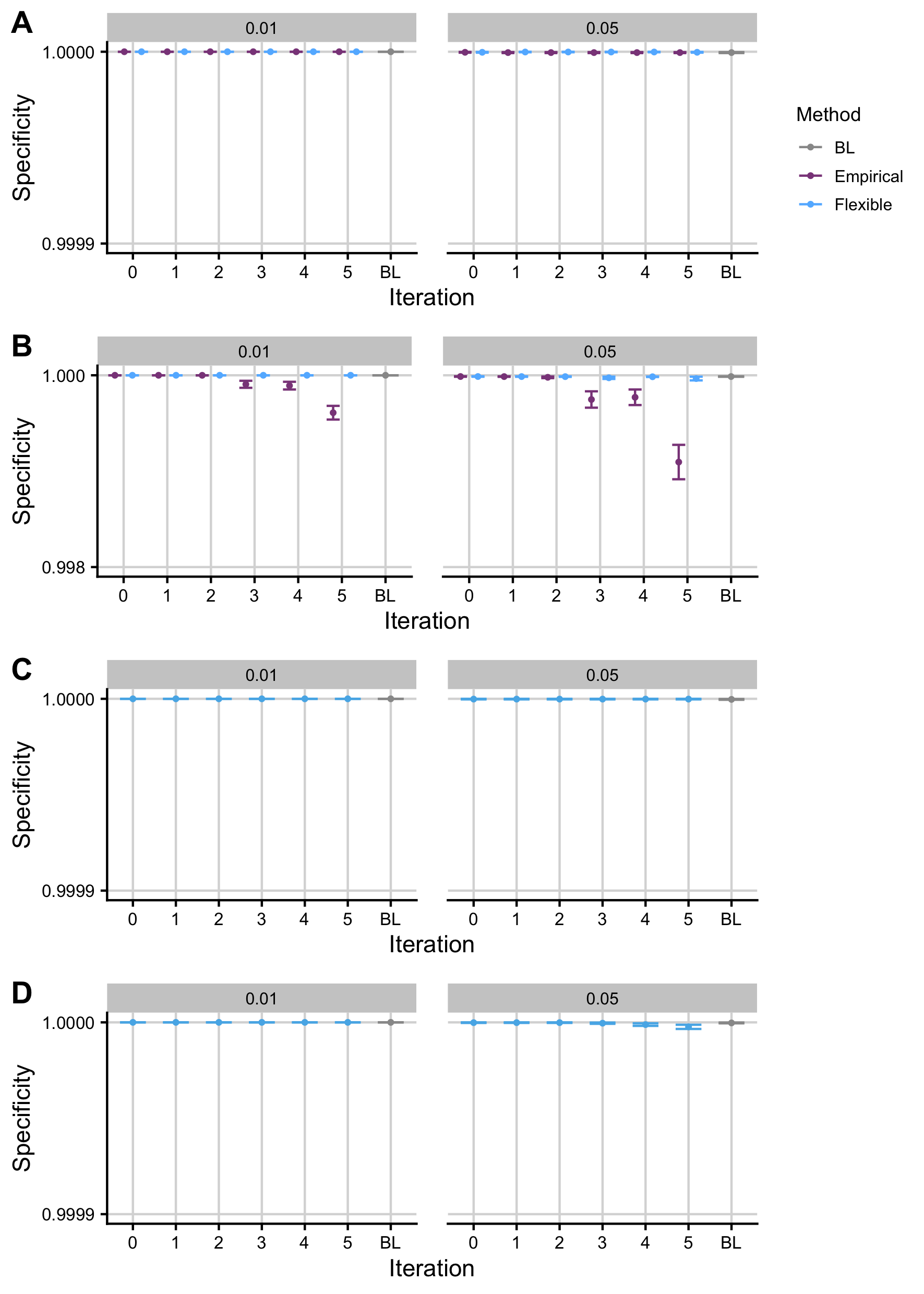

Supplement: S9 Fig — Mean +/- standard error for the specificity of FDR values from empirical and Flexible cFDR when iterating over independent (A; “simulation A”) and dependent (B; “simulation B”) auxiliary data that is bounded by [0, 1]. Panels C and D show the results from Flexible cFDR when iterating over independent (C; “simulation C”) and dependent (D; “simulation D”) auxiliary data simulated from bimodal mixture normal distributions. BL refers to results when using Boca and Leek’s FDR regression to leverage the 5-dimensional covariate data. Iteration 0 corresponds to the original FDR values. Our specificity proxy is calculated as the proportion of SNPs with r2 ≤ X with all the causal variants (“truly not-associated”), that were not detected with a FDR value less than 5 × 10^−6^, where results are faceted for X = 0.01, 0.05. Results were averaged across 100 simulations. (TIF) [file pgen.1009853.s009.tif]

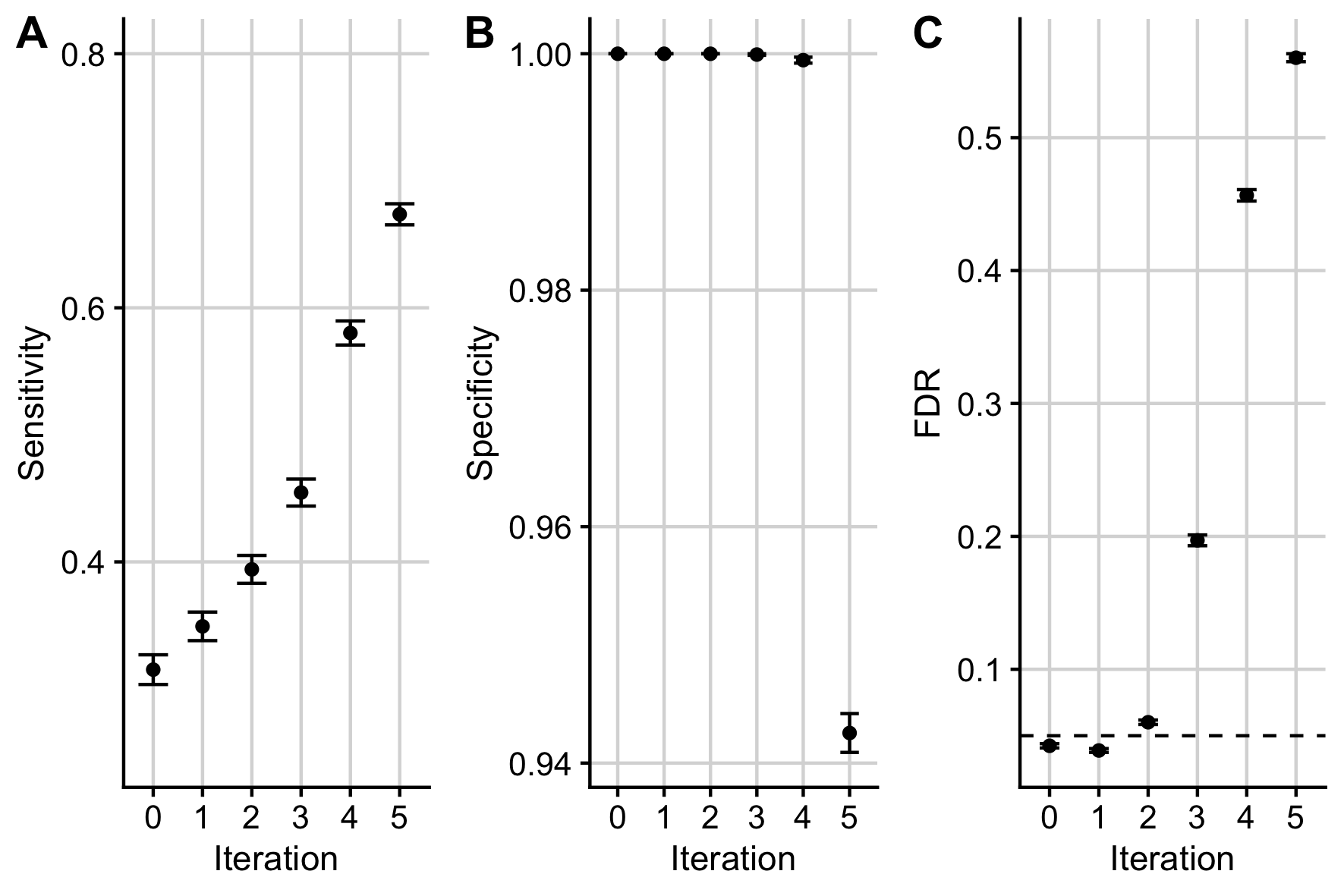

Supplement: S10 Fig — Mean +/- standard error for the sensitivity (A) specificity (B) and FDR (C) of FDR values from Flexible cFDR when iterating over the same dependent auxiliary data (“simulation E”). Iteration 0 corresponds to the original FDR values. Our sensitivity proxy is calculated as the proportion of SNPs with r2 ≥ 0.8 with a causal variant (“truly associated”), that were detected with a FDR value less than 5 × 10^−6^. Our specificity proxy is calculated as the proportion of SNPs with r2 ≤ 0.01 with all the causal variants (“truly not-associated”), that were not detected with a FDR value less than 5 × 10^−6^. Our FDR proxy is calculated as the proportion of SNPs that were detected with a FDR value less than 0.05, that had r2 ≤ 0.01 with all the causal variants (“truly not-associated”) (we raised α to 0.05 in order to assess FDR control within a manageable number of simulations). Results were averaged across 1000 simulations. (TIF) [file pgen.1009853.s010.tif]

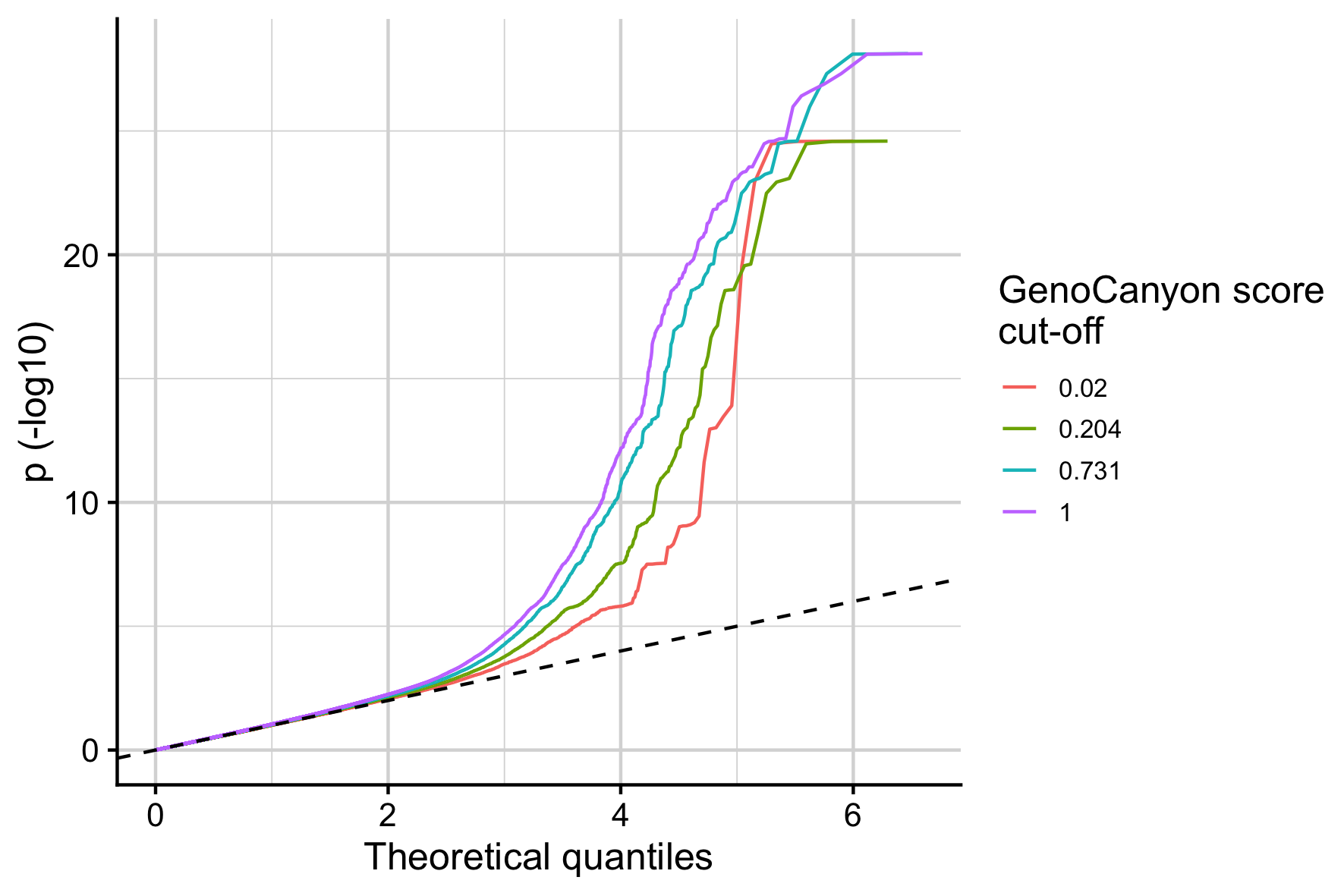

Supplement: S11 Fig — The values that were used to threshold the GenoCanyon scores are the quantiles of the distribution (0.020 is the 0.25 quantile, 0.204 is the 0.5 quantile, 0.731 is the 0.75 quantile and 1 is the maximum value). (TIF) [file pgen.1009853.s011.tif]

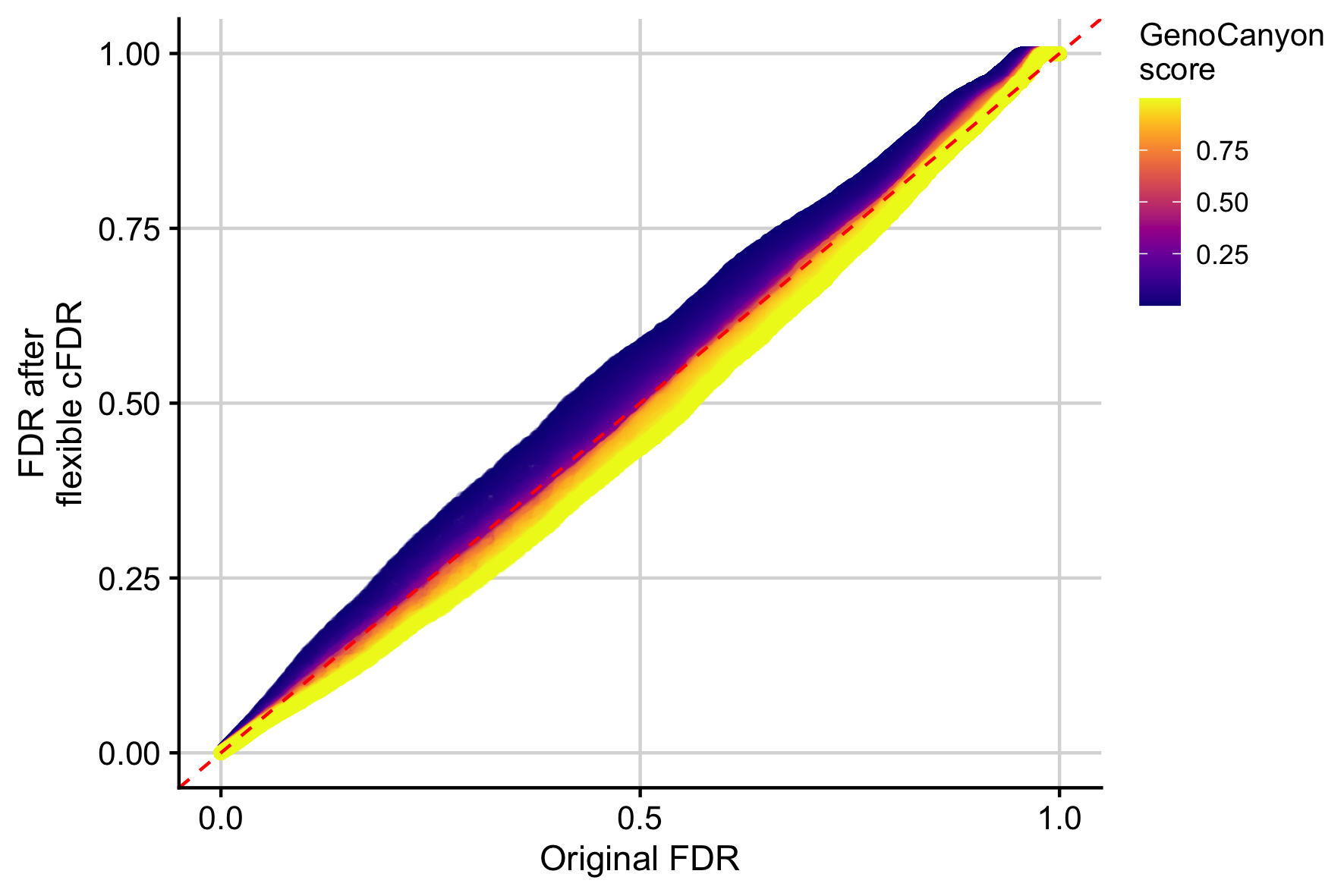

Supplement: S12 Fig — FDR values after using Flexible cFDR to leverage GenoCanyon scores with asthma GWAS p-values against raw FDR values coloured by GenoCanyon score. (TIF) [file pgen.1009853.s012.tif]

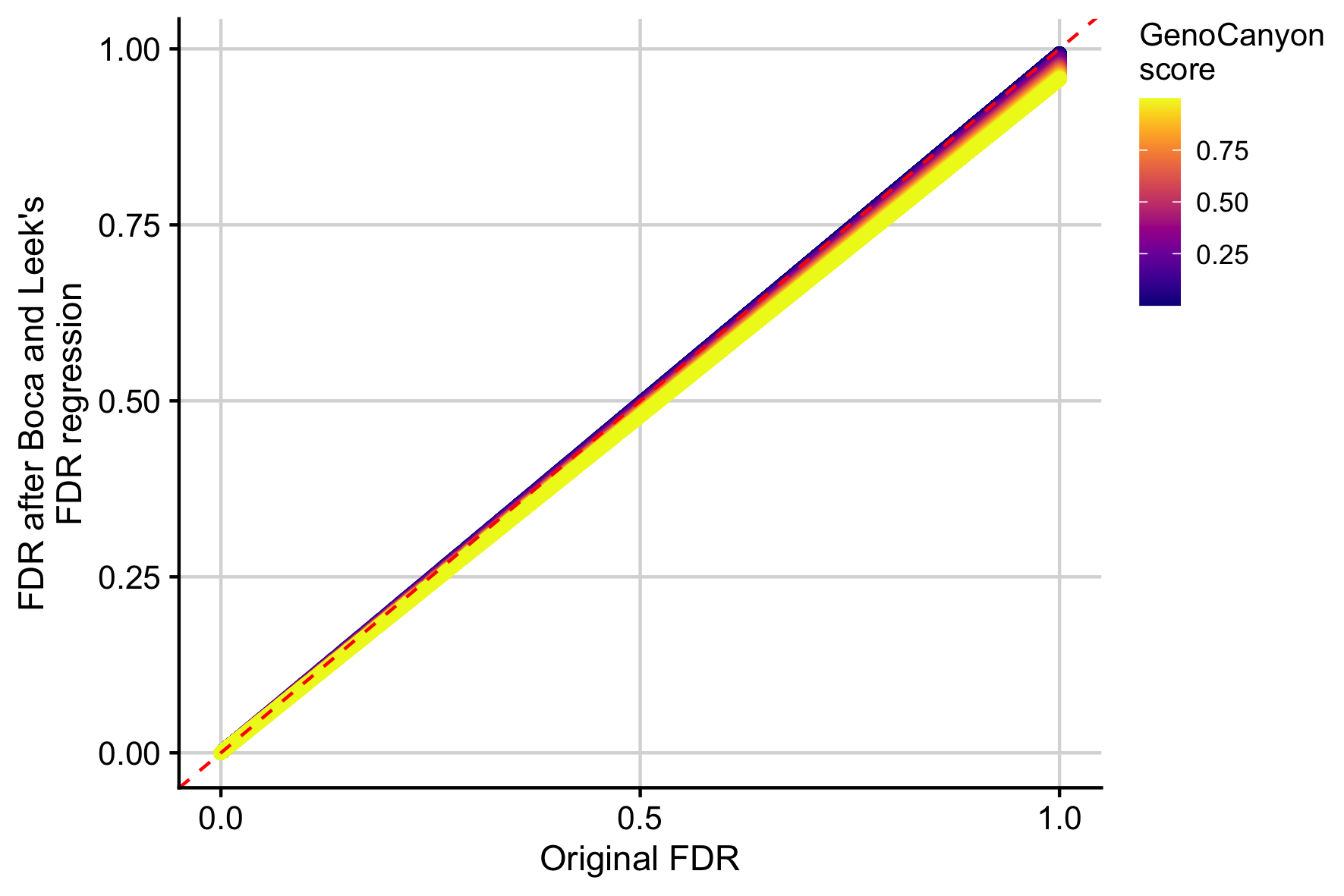

Supplement: S13 Fig — Adjusted p-values from BL when leveraging GenoCanyon scores with asthma GWAS p-values against raw FDR values coloured by GenoCanyon score. (TIF) [file pgen.1009853.s013.tif]

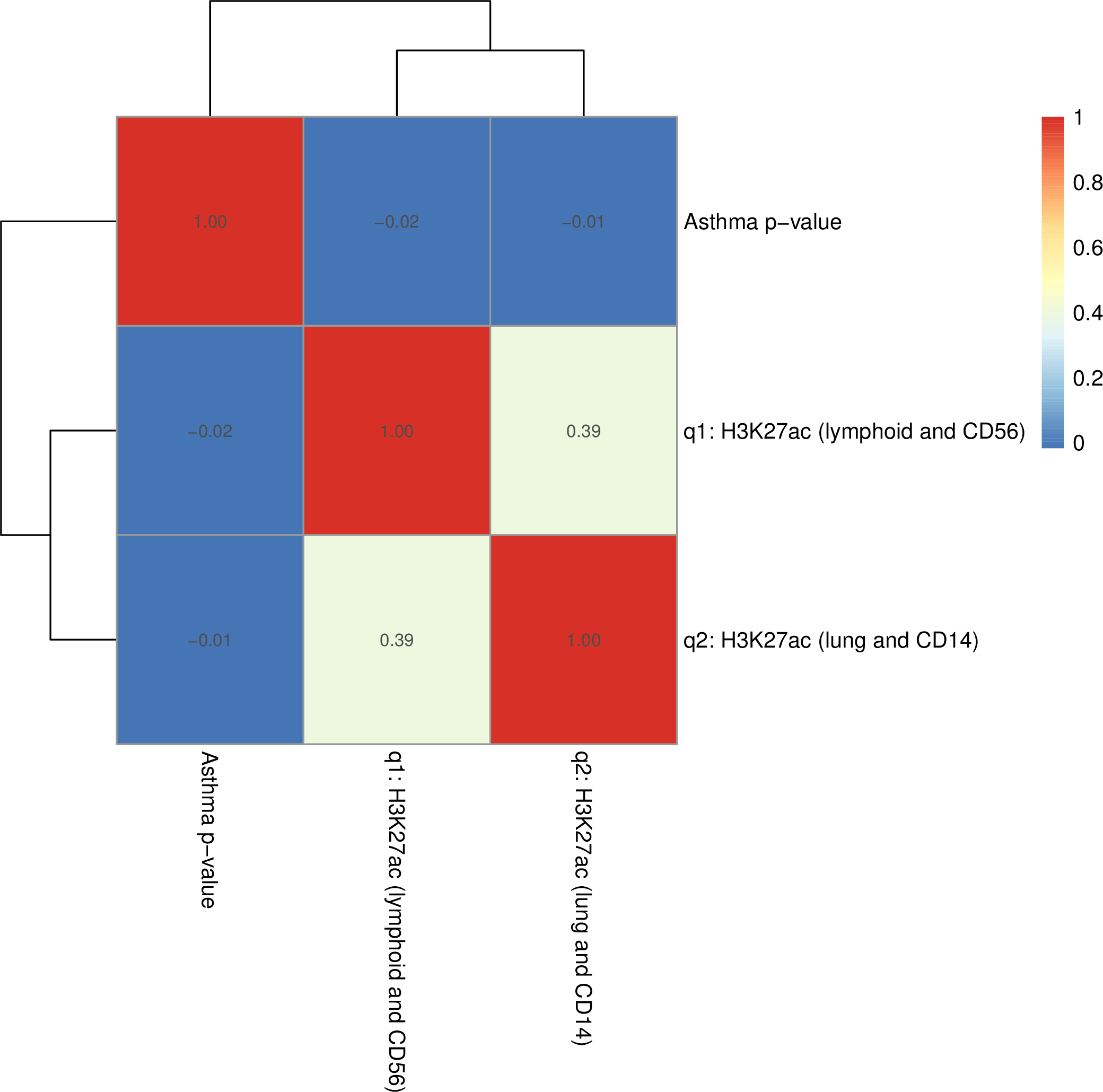

Supplement: S14 Fig — q1 is the average of (log transformed) H3K27ac fold change values in lymphoid and CD56 cell types. q2 is the average of (log transformed) H3K27ac fold change values in lung tissue and CD14+ cells. (TIF) [file pgen.1009853.s014.tif]

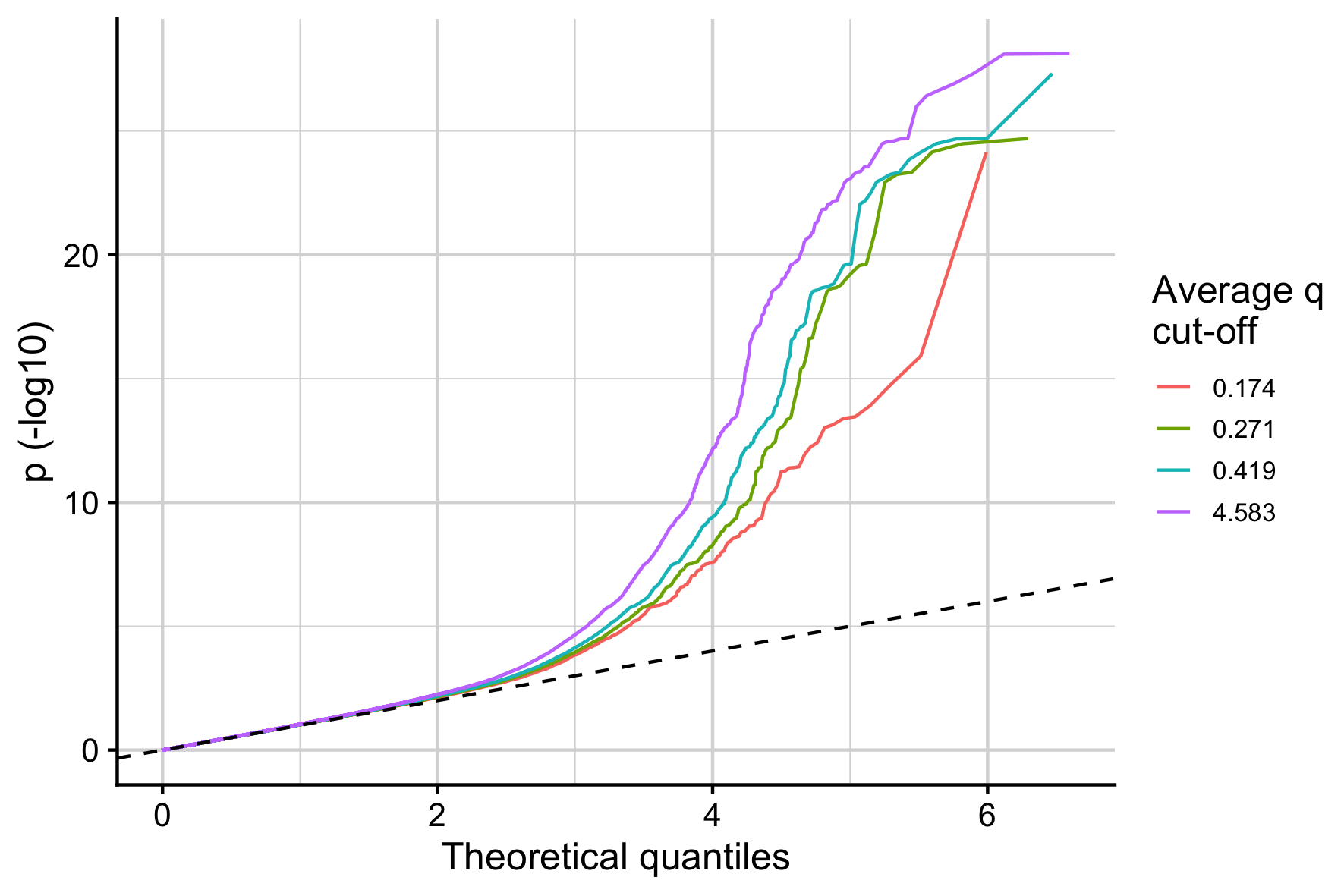

Supplement: S15 Fig — The values that were used to threshold q (average H3K27ac fold change values) are the quantiles of the distribution (0.174 is the 0.25 quantile, 0.271 is the 0.5 quantile, 0.419 is the 0.75 quantile and 4.583 is the maximum value). (TIF) [file pgen.1009853.s015.tif]

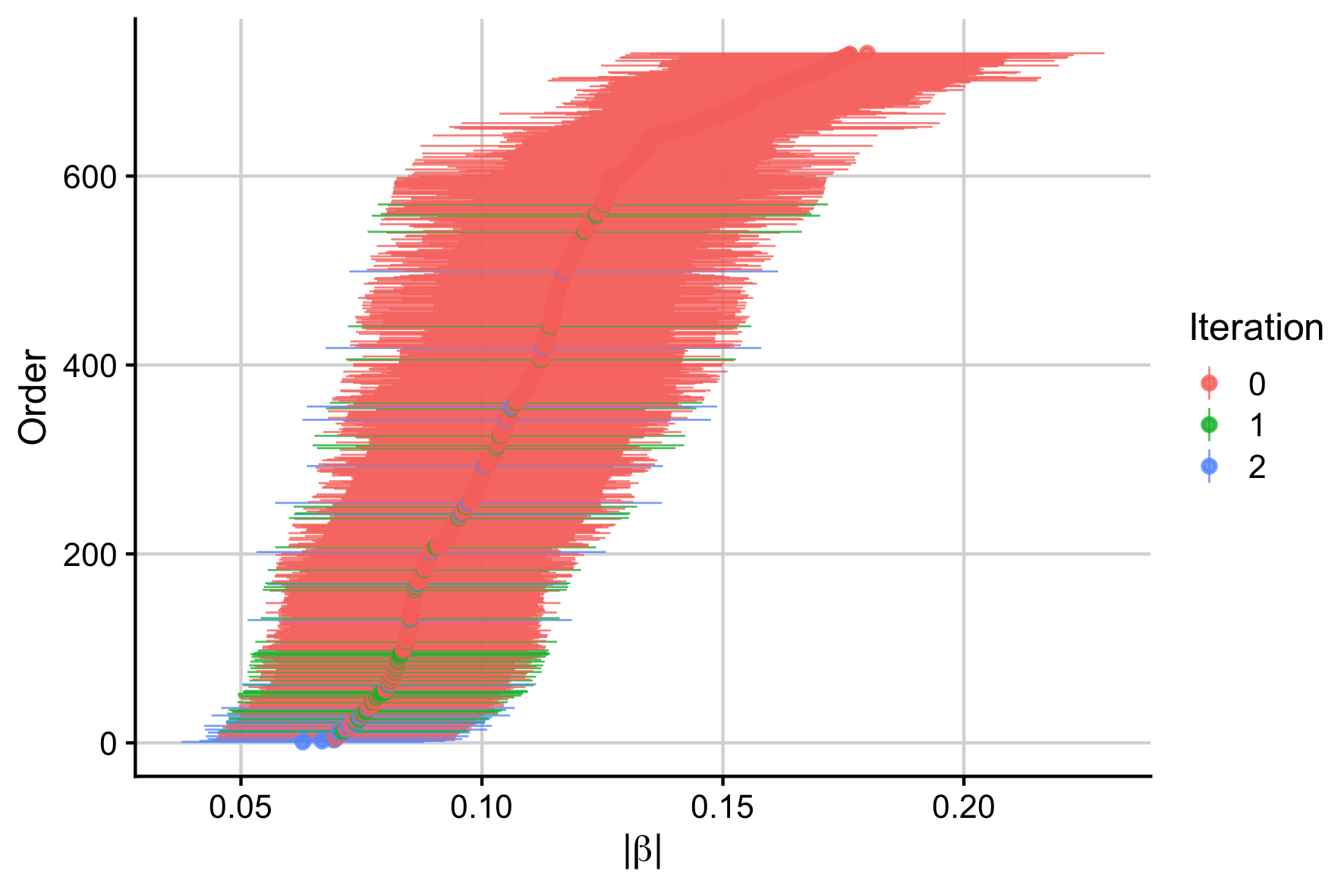

Supplement: S16 Fig — Absolute estimated effect sizes (|β|; log OR) +/ −1.96 × SE of SNPs significantly associated (FDR ≤ 0.000148249) with asthma in the original discovery GWAS data set (“iteration 0”) and those newly significant after iteration 1 and 2 of cFDR. (TIF) [file pgen.1009853.s016.tif]

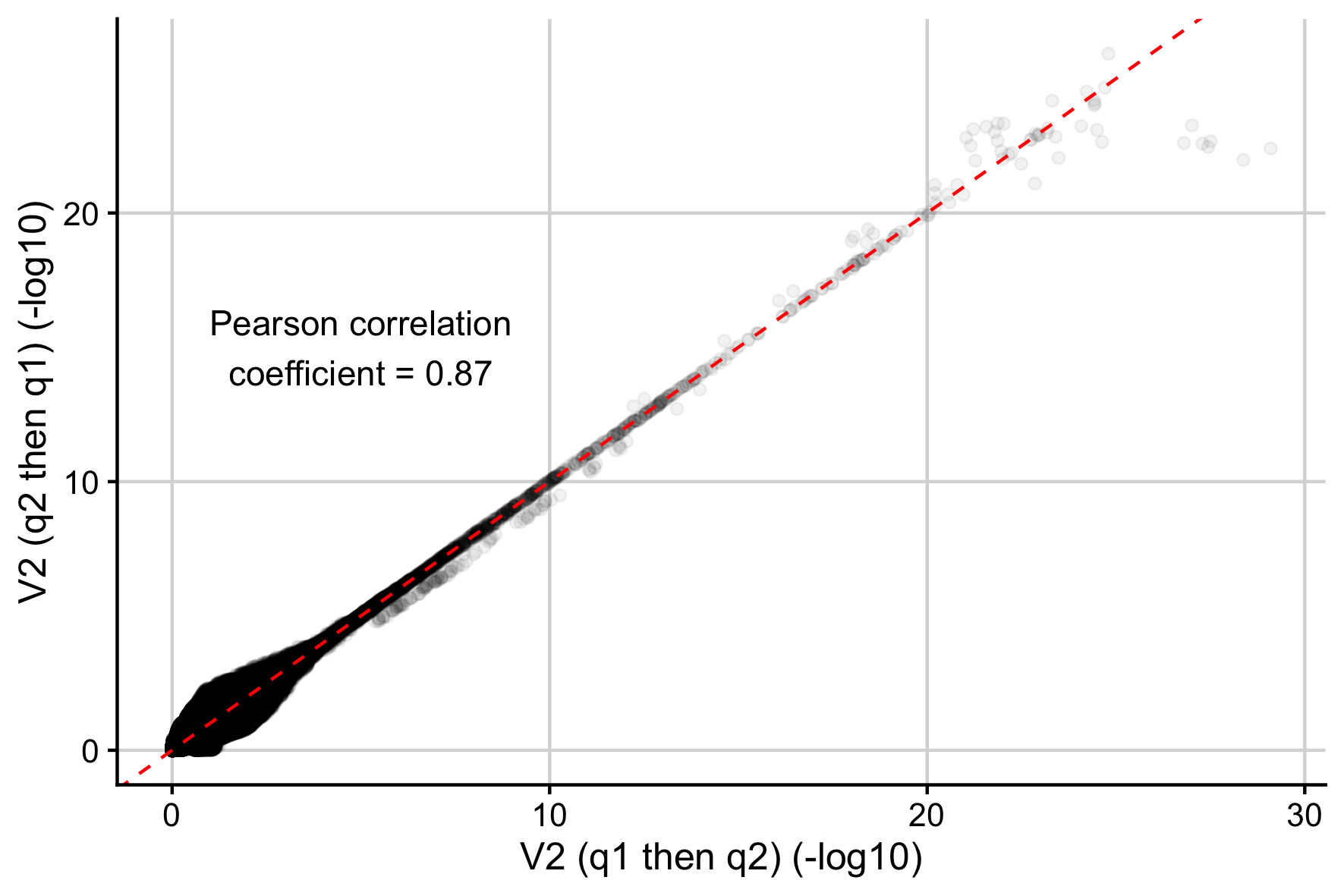

Supplement: S17 Fig — (-log10) v-values after 2 iterations of Flexible cFDR leveraging H3K27ac data when iterating over q2 and then q1 against (-log10) v-values when iterating over q1 then q2. (TIF) [file pgen.1009853.s017.tif]

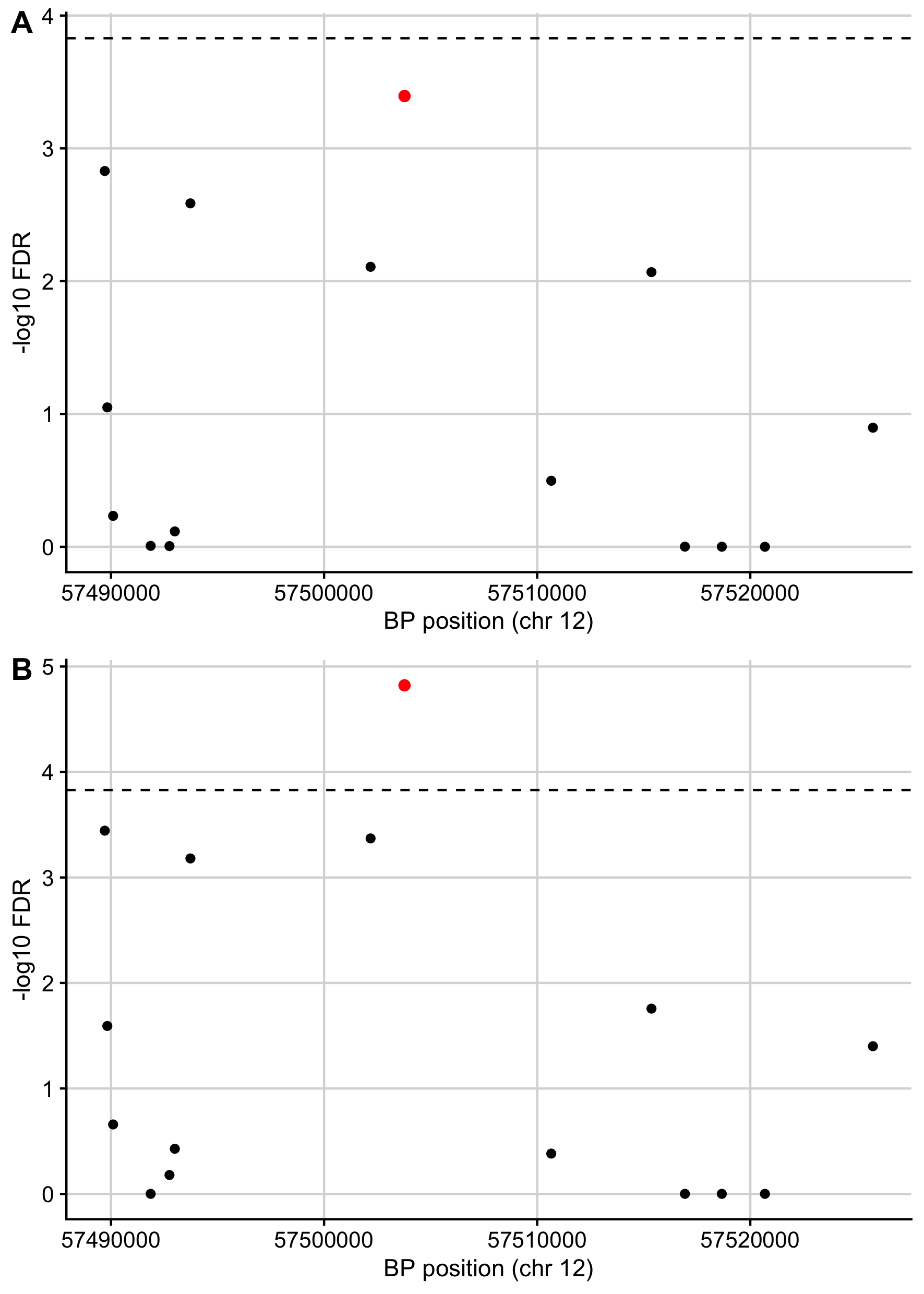

Supplement: S18 Fig — Manhattan plots of FDR values before (A) and after (B) applying Flexible cFDR for the region containing the STAT6 gene (chr12:57489187–57525922). Black dashed line at FDR significant threshold. Red SNP is rs167769 (index SNP). (TIF) [file pgen.1009853.s018.tif]

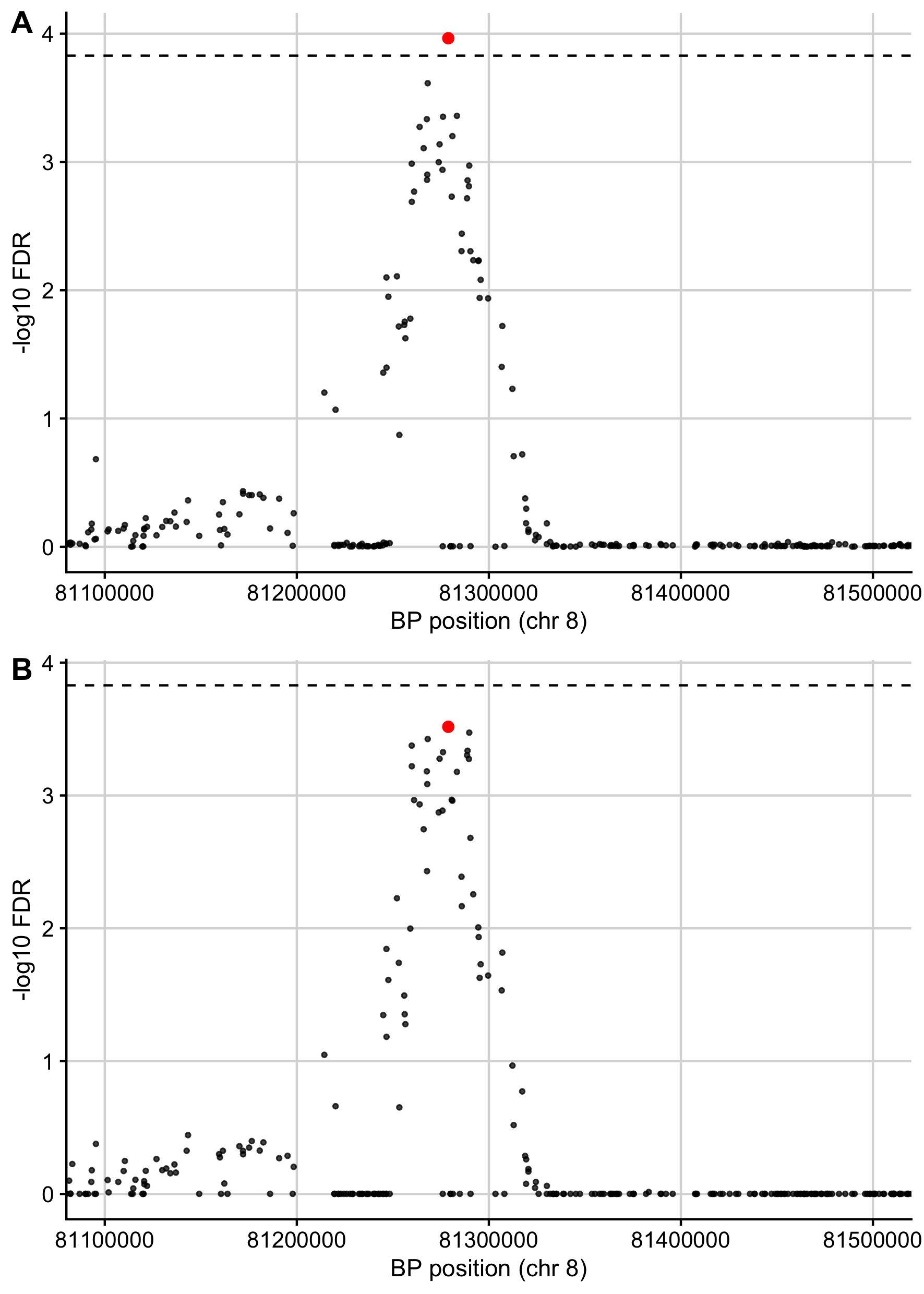

Supplement: S19 Fig — Manhattan plots of FDR values before (A) and after (B) applying Flexible cFDR for the region (chr8:81100000–81500000) containing index SNP rs12543811 (chr6:81278885) that is no longer FDR significant when applying Flexible cFDR. (TIF) [file pgen.1009853.s019.tif]

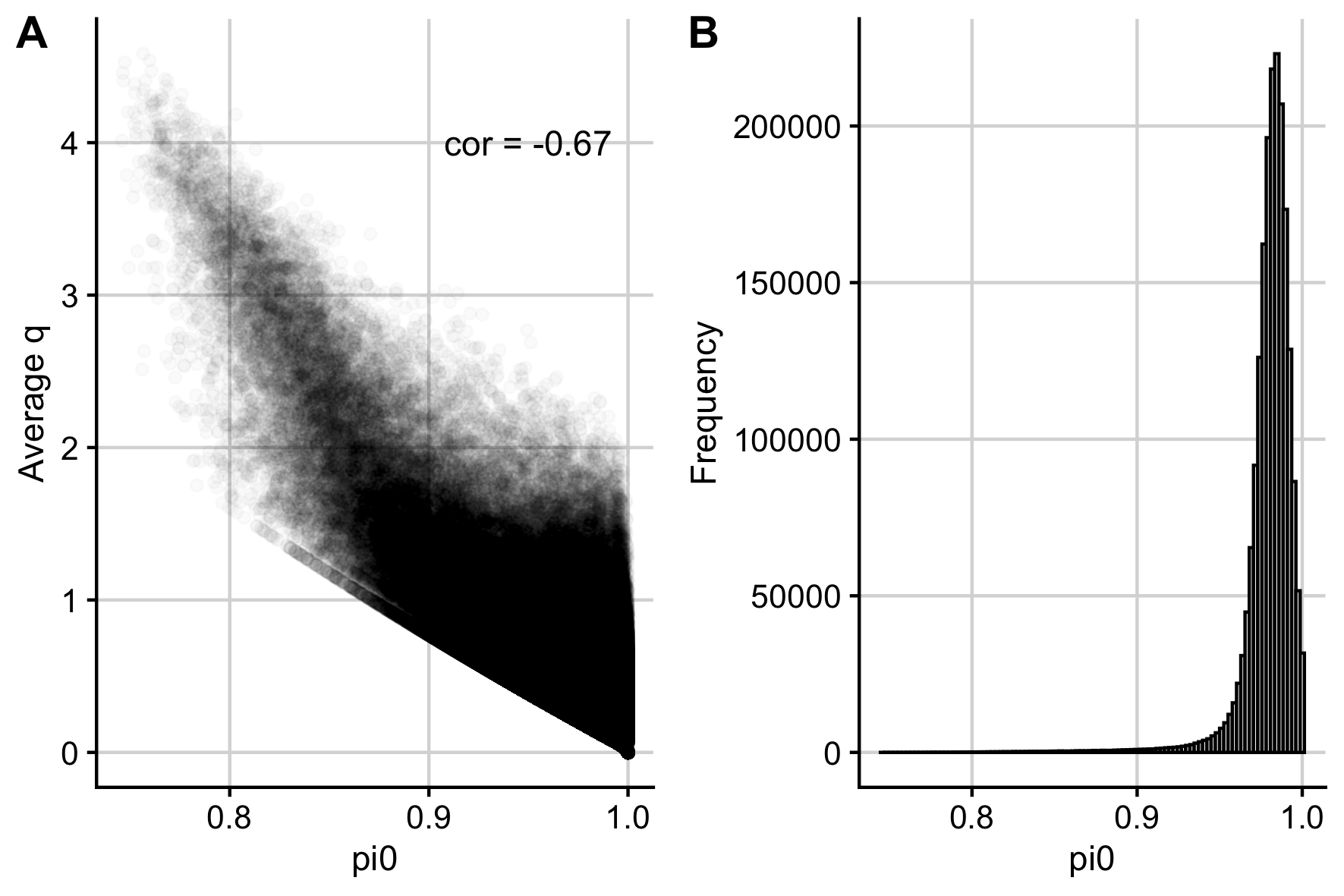

Supplement: S20 Fig — (A) Average H3K27ac fold change values in asthma relevant cell types (q) against estimated probabilities that the null hypothesis is true (‘pi0’) (B) Histogram of pi0 values for all 1,968,651 SNPs. (TIF) [file pgen.1009853.s020.tif]
