# Supplementary material for: Leveraging auxiliary data from arbitrary distributions to boost GWAS discovery with Flexible cFDR
Source: PLoS Genet. 2021 Oct 20;17(10):e1009853. doi: 10.1371/journal.pgen.1009853 (PMC8559959; doi:10.1371/journal.pgen.1009853)
Supplement: Attachment — Submitted filename: review-response.pdf [file pgen.1009853.s021.pdf]

Reviewer #1

In this paper the authors develop methods to estimate a form of false discovery rate in GWAS which conditions on auxilliary data that can have more flexible forms than previously proposed methods. They derive a "v-value" associated with the conditional FDR which can be used like a p-value in existing error control methods, and interestingly can be used iteratively with further sources of auxilliary data. The authors apply the approach to asthma data, revealing genetic associations with plausible mechanisms that are thoroughly researched. Overall I thought this was an excellent paper, written to a high standard, providing substantive advances in both analysis methodology and biological knowledge of asthma. Some minor comments follow.

We thank the reviewer for noting that our work provides substantive advances in methodology and biological knowledge of asthma.

1. line 80 the letter P gets used in multiple ways; on this line denoting "principal" in the null hypothesis as well as the random P-value, and elsewhere to denote probability. In the null hypothesis notation it sometimes occurs in upper-case and sometimes in lower-case (in equation 4, as both)

To avoid confusion, we have now updated the manuscript to use Pr(...) to denote probability rather than P(...). We have also corrected the null hypothesis notation so that p only appears in lower-case, we thank the reviewer for this observation.

In our new submission, the letter P is only used to denote “principal” in the null hypothesis and the random P-value. This is the notation used in the existing cFDR literature, such as Liley and Wallace (2021; Biometrical Journal), and we chose to maintain consistent notation in order not to confuse readers.

2. line 111 notwithstanding the flexibility in the kernel density estimation, your approach must work better for auxilliary data on a certain scale. Is it possible to comment on the best form of the auxilliary data?

The reviewer raises an interesting point and intuitively, the scale of the auxiliary data must matter. This will include the usual concerns about KDE; for example accuracy of kernel density estimates can be poor proximal to any hard boundaries on the data support or in regions of sparse data. Since there are numerous forms of auxiliary data that can be leveraged by Flexible cFDR, we have not explored in detail what an optimal form might be for arbitrary Q, and indeed it is likely to depend on the relationship between P and Q at the non-null SNPs. We agree that it is of interest to consider the scale of the auxiliary data and in our new submission we have also added the following text to the Discussion, highlighting that some thought about the scale of auxiliary data is useful:

Line 877: *“One can see that the scale on which the auxiliary data is measured may impact the performance of Flexible cFDR. Usual concerns about KDE apply, including that fits may be poor if there are regions with very sparse data. The optimal scale for the auxiliary data is likely to depend on the relationship between the principal p-values and the auxiliary data, and is not something we have explored here, but as usual, data visualisation is likely to be helpful to confirm that the scale for the auxiliary data is sensible. By default, the Flexible cFDR software returns a plot of the fitted 2D KDE and the estimated density of the auxiliary data overlaid onto the real data values, enabling users to visually examine the fit of the KDE to their data.”*

3. line 112 "convert the p-value" - how to deal with 2-tailed p-values?

The p-values for the principal trait that we refer to throughout the whole manuscript refer to those obtained from a GWAS, which are generally two-sided. As the sign of the associated Z score is essentially arbitrary (depending on which allele is designated “effect”), we transform the p-values to absolute Z scores. To fit the KDE, we then mirror the Z scores to the negative real line (together with their associated Q values) but extract a fitted KDE for only the non-negative part of the data. This avoids boundary effects which would otherwise bias estimates near 0.

This is an important technical detail that we did not explain fully in our initial submission. In our new submission we have updated the Methods section to better describe this process:

Line 161: *“To estimate both Pr(Q\leq q|H_0^p) and Pr(P\leq p, Q\leq q) in Eq 5 we first fit a bivariate kernel density estimate (KDE) using a normal kernel. To do this, we transform the p-values for the principal trait (derived from a two-tailed test, as is typical in GWAS) to absolute Z-scores (Z_p; since the sign of the associated Z-scores are essential arbitrary as they depend on which allele is designated ``effect’). To avoid boundary effects which would otherwise bias estimates near 0, we mirror the absolute Z-scores onto the negative real line together with their associated Q values but only estimate the KDE on the non-negative part of the data, consequently modelling the PDF corresponding to Z_p, Q in the usual way as…”*

4. equation 9, the denominator suggests that you assume strong evidence to be large z and small q. Would this be modified for auxilliary data with large values of q providing stronger evidence?

The cFDR estimator does assume that larger z are enriched for smaller q and this relates to the later point on stochastic monotonicity (reviewer #1 comment 7). As suggested by the reviewer, this means auxiliary data where larger z are instead enriched for larger q must be modified. We do this by flipping the sign of the auxiliary data values such that larger z are now enriched for smaller q. The Flexible cFDR software automatically calculates the correlation between p and q and flips the sign of q if necessary. This is exemplified in our two real-data analyses whereby the relationship between p and q is negative since small asthma GWAS p-values are enriched for larger GenoCanyon scores (application 1) and larger H3K27ac fold change values in asthma relevant cell types (application 2). In our new submission, we highlight that Flexible cFDR flips the sign of the auxiliary data in these instances.

5. line 136 change the second "and" to "but"

This change has been made.

6. line 153 "analogous to that of a p-value" - some more detail would be helpful: it's not obvious that we can just plug these into an FDR algorithm. First we have to realise that v-values are uniformly distributed (in the sense that P(v<a)=a) under H0P. I think I can see this intuitively, but it would be nice to have this explained (or even better, a formal proof).

In our new submission, we explain that v-values are analogous to p-values in that they share the property that they are uniform on $[0, 1]$ under the null hypothesis, and we point readers to the mathematical proof of this outlined in Liley and Wallace (2021; Biometrical journal):

Line 216: *"The v-value can be interpreted as the probability that a randomly-chosen (p,q) pair has an equal or more extreme cFDR value than cFDR(p_i,q_i) under H_0^p and are thus analogous to p-values. We refer readers to Theorem 3.1 and its accompanying proof in Liley and Wallace (2021) which shows that the v-values are uniformly distributed under the null hypothesis for X=(p_i,q_i)\in [0,1]^2, and this naturally holds for Flexible cFDR where X=(p_i,q_i)\in [0,1] \times [q_{low}, q_{high}] (where q_{low} and q_{high} are the lower and upper limits of the KDE support respectively)."*

7. line 159 "arbitrary" - not quite as you state elsewhere that the distribution should be monotonic in p. Can you define this monotonic property somewhere as in practice it can only be a stochastic property?

We now use the terminology "positive stochastic monotonicity" to describe the relationship between p and q, and use this consistently throughout our manuscript:

Line 121: *"The cFDR framework implicitly assumes that there is a ``positive stochastic monotonic relationship'' between p and q, meaning that on average SNPs with smaller p-values in the conditional trait are enriched for smaller p-values in the principal trait. This assumption is naturally satisfied in the typical use-case of cFDR that leverages p-values for genetically related traits."*

We have re-written the specific sentence mentioned by the reviewer (originally line 159, now line 210) to read: "*In the original method, f_0(p,q) is estimated using a mixture-Gaussian distribution, but to support auxiliary data from arbitrary distributions (specifically, where the only distributional constraints for the auxiliary data is that it is positively stochastically monotonic in p) we utilise the assumptions in Eq 2 to write f_0(p,q)=f_0^q(q) (since the PDF of p conditional on H_0^p is the standard uniform density).*".

8. Figure 1 and elsewhere, the dashed line shows specificity 1-5e-6, presumably as a reference to the FDR of 5e-6. But 1-spec is the false positive rate, not the FDR. The dashed line may be confusing, especially as it tends not to match the actual specificity. Note also that the manuscript uses two different notations for small numbers.

We agree that the dashed line showing the false positive rate in Figures 1 and 2 was confusing and have now removed it.

In our new submission, we now explicitly calculate an FDR proxy in our simulation analysis and have updated Figure 1 and Figure 2 to examine FDR control (observing that Flexible cFDR controls the FDR whereas empirical cFDR and Boca and Leek's FDR regression do not in the simulated scenarios we consider).

Line 348: *"To assess whether the FDR was controlled within a manageable number of simulations, we raised \alpha to 0.05 and calculated the proportion of SNPs called FDR significant which were truly not-associated (that is, r^2<=0.01 with all of the causal variants)."*

We have now carefully checked the manuscript to ensure that all values are in scientific format, displaying the numbers in exponential notation (i.e. 1e-06 rather than 1×10^{-6}).

9. line 562 "55 newly significant SNPs" - how does this square with 118 SNPs earlier in the sentence?

In our new submission we have carefully rechecked all SNP counts to ensure that they are correct.

10. line 665 "course" -> "coarse"

This has been corrected.

11. reference 11, journal name is incomplete.

This has been corrected.

Reviewer #2

The authors introduce an extension to the cFDR framework for incorporating auxiliary information from any arbitrary distribution to improve power for detecting GWAS associations. The authors demonstrate their approach via simulations and leverage functional information such as GenoCanyon scores for detecting asthma GWAS associations. However, there are several concerns below regarding the novelty and contribution of the manuscript listed below.

We thank the reviewer for providing an accurate summary of our manuscript and address the reviewer's concerns about the novelty and contribution of the manuscript below.

Major Concerns:

Contribution: The authors' manuscript relies heavily on a preprint by Liley and Wallace (2021) and is presented as a necessary extension to account for continuous covariate. However, the heavy reliance on Liley and Wallace (2021) appears to weaken this manuscript's contribution.

The reviewer's concerns about the contribution of our manuscript suggest that in our initial submission we did not effectively communicate the novelty of our method and it's gains over the conventional cFDR approach. In our response below we outline (i) the key insights which we transferred from the conventional approach, (ii) the methodological and practical advances of our method and (iii) the quantitative gains of our method over the conventional approach.

Liley and Wallace (2021; now in Biometrical Journal https://doi.org/10.1002/bimj.201900254) describe an empirical cFDR method and prove several key properties, in particular that it can control the FDR in high dimensional hypothesis testing with covariates. The key insights in enabling the approach are (1) that the estimated cFDR can be used to order and map points in the 2D $p \times q$ unit square to a 1D unit line, and (2) that these remapped values are uniform on $[0, 1] \mid H_0^p$. However, the estimation of cFDR in this approach combines empirical estimates with an assumption that the covariates can be transformed to a mixture of centred normals, which is appropriate for GWAS p-values but not for other covariates such as functional genomic data. Our manuscript builds on Liley and Wallace (2021) for an altogether different purpose – that is to leverage functional genomic data

with GWAS test statistics - by defining a new estimator. Indeed, the cFDR approach has never before been used in the functional genomics context.

To adapt the method for the functional genomics setting, our manuscript describes four key advances. Firstly, we derive an estimator based on a 2-dimensional KDE of the bivariate distribution rather than empirical estimates, which is considerably faster. Secondly, both approaches require the estimation of $q|H_0^p$, which Liley and Wallace approximate by $q|p > 1/2$. In contrast, Flexible cFDR utilises the local FDR to empirically evaluate the influence of specific p-value quantities on the null hypothesis and uses these in the estimation of $q|H_0^p$. Thirdly, we remove the assumption that $q|H_0^p$ can be transformed to a mixture of centred normals, and instead integrate over the KDE estimated earlier on, which relaxes the distributional assumptions placed on q. Fourth, Flexible cFDR is supported by user-oriented software which is thoroughly documented in an easy-to-navigate website (https://annahutch.github.io/fcfdr/) and which includes several fully reproducible vignettes – thus enabling a wider range of researchers to explore the cFDR approach in their work.

In our original submission, we showed that Flexible cFDR outperformed the empirical approach in terms of specificity and speed. In our new submission, we additionally show that Flexible cFDR outperforms the empirical approach in terms of FDR control (Fig 1A, Fig 1B). This, combined with the fact that Flexible cFDR removes all parametric assumptions placed on the auxiliary data, suggests that it could supersede earlier empirical approaches.

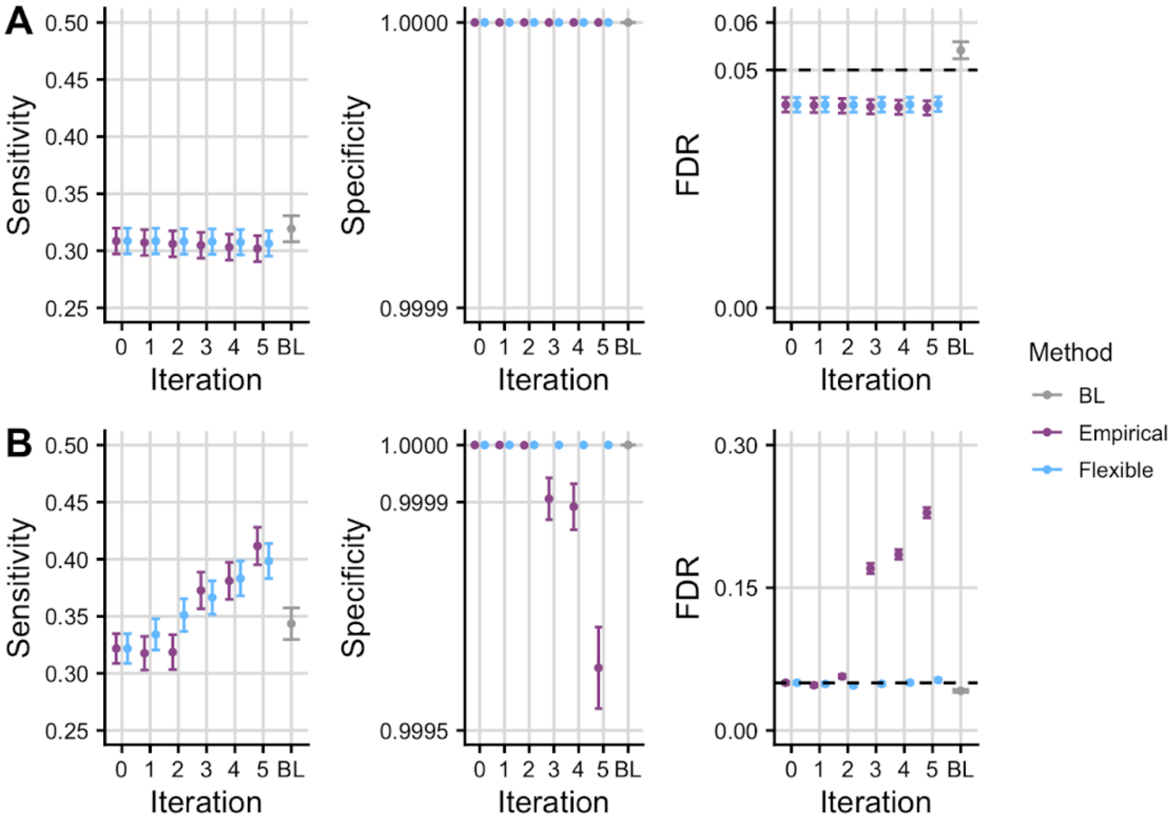

Motivated by the reviewer's comment, we have re-structured and re-written parts of the Methods section to better highlight the novel contributions of our manuscript. In particular, we begin by restating the definition and empirical estimator of the cFDR (in the subsection "Conditional false discovery rate") before describing our novel methodological advances in the "Flexible cFDR" subsection. We have also explicitly listed the advances of Flexible cFDR over earlier empirical approaches in the Discussion section:

Line 862: *"Our manuscript describes four key advances enabling the extension of the cFDR framework to the functional genomics setting. Firstly, we derive an estimator based on a 2-dimensional KDE of the bivariate distribution rather than empirical estimates, making our method considerably faster than earlier empirical approaches. Secondly, the cFDR framework requires the estimation of q|H_0^p, which Liley and Wallace (2021) \cite{liley2021} approximate by q|p>1/2. In contrast, Flexible cFDR utilises the local FDR to empirically evaluate the influence of specific p-value quantities on the null hypothesis and uses these in the estimation of q|H_0^p. Thirdly, we remove the assumption that q|H_0^p can be transformed to a mixture of centred normals, and instead integrate over the KDE estimated earlier on, which relaxes the distributional assumptions placed on the auxiliary data. Fourth, Flexible cFDR is supported by user-oriented software which is thoroughly documented in an easy-to-navigate website (\url{https://annahutch.github.io/fcfdr/}) which includes several fully reproducible vignettes illustrating how Flexible cFDR can be implemented with a desired error rate on a particular data set of interest - thus enabling a wider range of researchers to explore the cFDR approach in their work."*

Additionally, the authors' literature review understates the availability of flexible methods for incorporating auxiliary information. There are a number of additional methods explored in a review article by Korthauer et al. (2019) that already allow for flexible types of covariates while providing guaranteed FDR control at the user's desired error rate. For instance, on page 3 the authors cite Lei and Fithian (2018) but incorrectly state that "the underlying parametric assumptions may not may not be satisfied, resulting in the loss of statistical guarantee." This is an incorrect statement. In fact, one of the strengths of Lei and Fithian (2018) is that the modeling of side information can be misspecified, yet, as long as assumptions regarding the distribution of null p-values is maintained, finite sample FDR control is guaranteed. Model misspecification only affects the power. Furthermore, Lei and Fithian (2018)'s approach has been successfully demonstrated in a GWAS setting with high-dimensional auxiliary information (Yurko et al. 2020). A more comprehensive comparison to these existing methods is warranted for properly assessing the authors' contributions.

The reviewer raises concerns about the insufficient discussion of, and comparison with, existing methods in the literature in our initial submission. We regret that our initial literature review was incomplete and our inclusion of an incorrect statement regarding Lei and Fithian's AdaPT method. In our new submission, we have rewritten the Introduction and the Discussion to better describe the existing literature (additionally removing our incorrect statement about AdaPT).

In our initial submission we compared Flexible cFDR to GenoWAP and FINDOR, but we agree that additional methods were missing from this comparative analysis, particularly those from the statistical literature of covariate based multiple testing. We thank the reviewer for highlighting the benchmarking paper by Korthauer et al. (2019) which provides a detailed comparison of eight related methods, including the standard BH and q-value approaches. We consider the six remaining covariate-based frameworks in turn. Firstly, the adaptive shrinkage (ASH) method is not directly relevant as it takes as input only an effect size estimate and corresponding standard error. Secondly,

both IHW and Cai and Sun's Conditional Local FDR (LFDR) approach bin data based on covariate values, and FINDOR (which we already use for comparison) is shown to be superior to IHW (in terms of false positive findings) in the FINDOR manuscript. For completeness, we now additionally compare Flexible cFDR with IHW as an exemplar of binning approaches. We do this for the GenoCanyon analysis since it is not clear how to apply IHW to multi-dimensional covariates, as is the case for the ChIP-seq analysis.

The remaining three methods described in Korthauer et al. (2019) are FDR regression (Scott et al. 2015), Boca and Leek's FDR regression (BL) and AdaPT (Lei and Fithian 2018). Since Boca and Leek's FDR regression is similar to, but outperforms Scott et al.'s FDR regression (in terms of gains in true positive rate in Korthauer et al. 2019), in our new submission we include a direct comparison of Flexible cFDR with Boca and Leek's FDR regression in both our simulation-based analysis and the GenoCanyon and the ChIP-seq asthma applications.

Unfortunately, we were unable to run Lei and Fithian's (2018) AdaPT on our asthma data due to its computational demands. The documentation warns of "ultra-large scale problems (n > 10^5)" but in our case n is approximately 2 million. Consequently, we attempted to run AdaPT on our independent subset of approximately 500,000 SNPs with our two-dimensional covariate vector, but this still caused the software to crash. In Yurko et al. (2020), where AdaPT was used in the GWAS context, the authors included a pre-selection step to identify likely functional SNPs and ran AdaPT only on this subset of SNPs. This step reduced their number of SNPs from 1,109,226 to just 25,076 SNPs (a 97.7% reduction). We chose not to include a pre-selection step in our analysis as this would make the findings difficult to compare. Indeed, one of the benefits of cFDR is that it is computationally efficient enough to be run genome-wide.

In all, we now compare Flexible cFDR to GenoWAP, IHW, Boca and Leek's FDR regression (BL) and FINDOR. The following paragraph in the Discussion summarises the results of from these comparisons:

Line 811: "*We compared the performance of Flexible cFDR to that of four comparator methods which have previously been shown to outperform other approaches \cite{korthauer2019, kichaev2019}: GenoWAP, IHW, BL and FINDOR. Whilst also intending to compare our method to AdaPT \cite{lei2018}, this approach uses a p-value masking procedure which takes many iterations of optimisation and can be computationally expensive \cite{zhang2019}. We found AdaPT to be too computationally demanding for large-scale GWAS data and previous studies suggest that a SNP pre-filtering stage is required \cite{yurko2020}. Of the methods considered, we found that only BL was as versatile as Flexible cFDR. Specifically, IHW currently only supports univariate covariates and, unlike Flexible cFDR, cannot be applied iteratively to leverage multi-dimensional covariates because it outputs FDR-adjusted p-values derived using an optimal weighting procedure (rather than raw p-values). In GenoWAP, the prior probability used in the model are calculated as the mean GenoCanyon score (or tissue-specific GenoSkyline \cite{lu2016a} or GenoSkyline-Plus \cite{lu2017} score) of the surrounding 10,000 base pairs, thereby restricting its utility to leveraging only these scores (which we found were unlikely to capture enough disease relevant information to substantially alter conclusions from a study). And finally, FINDOR bins SNPs based on how well they tag heritability enriched categories and this requires the estimation of \chi^2 statistics (i.e., tagged variance) for each SNP using a range of functional annotations, which are generally those in the baselineLD model \cite{gazal2017}. Users are thus required to run LD-score regression prior to running FINDOR, and this two-step approach may limit the accessibility of the method. Although BL*

was as versatile as Flexible cFDR, in a simulated-based analysis we found that it often failed to control the FDR and was less powerful than Flexible cFDR. Whilst FINDOR was shown to be the most powerful method, this may reflect the information gain in leveraging 96 annotations rather than a single histone mark. This emphasises the importance of being able to iterate over different auxiliary measures, and suggests that a fruitful area of extension for cFDR will be to increase the robustness of FDR control for dependent q."

Comments on robustness: The authors state on page 33 that their extension "provides a statistically robust framework". This is a strong statement to make given the limitations regarding the cFDR approach with desired error-rate control. Unlike the popular BH method for FDR control, the authors' approach requires the adjustments by Liley and Wallace (2021). It is not clear in the current manuscript how one can apply Flexible cFDR with a desired error rate to a particular dataset of interest. This is an important concern regarding the robustness of the approach.

The reviewer's concerns about the robustness of our approach shows that we did not effectively describe the process of using Flexible cFDR to identify associations with a desired error rate in our original submission. The key idea is that since the v-values from Flexible cFDR are analogous to p-values, sharing the property that they are uniform on [0,1] under H_0^p, these can be used directly in any error-rate controlling procedure (for example in the BH procedure to generate FDR values).

Motivated by the reviewer's concerns, in our new submission we now direct readers to a mathematical proof that v-values are uniform on [0,1] under the null and thus are analogous to p-values and can be used in any error-rate controlling procedure:

Line 216: "*The v-value can be interpreted as the probability that a randomly-chosen (p,q) pair has an equal or more extreme cFDR value than cFDR(p_i,q_i) under H_0^p and are thus analogous to p-values. We refer readers to Theorem 3.1 and its accompanying proof in Liley and Wallace (2021) which shows that the v-values are uniformly distributed under the null hypothesis for X=(p_i,q_i)\in [0,1]^2, and this naturally holds for Flexible cFDR where X=(p_i,q_i)\in [0,1] \times [q_{low}, q_{high}] (where q_{low} and q_{high} are the lower and upper limits of the KDE respectively)."*

We also now explicitly state that these can be used in any error rate controlling procedure:

Line 223: *"Deriving v-values, which are analogous to p-values, means that the output from Flexible cFDR can be used directly in any conventional error rate controlling procedure, such as the BH method \cite{benjamini1995}."*

Our two applications leveraging various auxiliary data with asthma GWAS p-values also illustrate how v-values are generated and then used in the BH-procedure to generate FDR values. In our new submission we have updated the relevant sections in the Methods and Results to better highlight this step. We also now refer readers to the accompanying software for our method which contains two completely reproducible vignettes exemplifying how Flexible cFDR can be applied to various datasets of interest with a desired error rate.

Line 246: *"We have created an R package, fcfdr (https://github.com/annahutch/fcfdr), that implements the Flexible cFDR method. The software web-page (https://annahutch.github.io/fcfdr/) contains fully reproducible vignettes which illustrate how the Flexible cFDR method can be used to generate*

v-values from GWAS p-values and covariate data, and how these can be used directly in any error rate controlling procedure (for example \pkg{p.adjust} with `method=``BH''' for BH-adjusted p-values)."

We agree that "a statistically robust framework" is a strong statement to make, but based on the mathematical foundations of the v-values and our new simulation analysis which shows that Flexible cFDR does maintain FDR control in the simulation scenarios considered, we believe that this is an authentic statement in this case. That being said, if the reviewers or editors would prefer that this statement was removed, then we are open to making this change.

Dependence: It is not clear from the manuscript how LD affects the performance of their proposed method. Given the prevalence of LD between SNPs in GWAS this is a critical issue.

Our initial submission did not effectively communicate that a major advantage of our method is that it is not affected by LD between SNPs, unlike comparator methods such as Boca and Leek's FDR regression where "control of the FDR is worse with increasing correlation" (Boca and Leek 2018). This is because the Flexible cFDR methodology uses an independent subset of SNPs for estimating the KDE. (Users must therefore supply an independent set of SNPs to the Flexible cFDR software but to make this as straightforward as possible, we have added an additional vignette to the Flexible cFDR webpage describing how to generate LDAK weights for SNPs (https://annahutch.github.io/fcfdr/articles/ldak-vignette.html)). Generated v-values will of course be positively correlated due to LD, but the Benjamini-Hochberg FDR approach is valid in cases of positive dependency. This important feature of Flexible cFDR is clarified in our new submission:

Line 842: *"Whilst LD between SNPs is often a concern (e.g. because methods such as KDE assume independence between observations), we fit the KDE to a subset of LD-independent SNPs, but then generate v-values for the full set of SNPs, thereby benefiting from computational efficiency but also facilitating downstream analyses which typically require the full set of SNPs, such as fine-mapping or meta-analysis. LD means that the v-values will be positively correlated, so we appeal to the established robustness of the BH FDR estimation to positive dependency \cite{benjamini2001}."*

In exploring this step in more detail, we noticed that the MAF distribution may differ between the LD-independent subset and the full dataset (because rare SNPs are generally in LD with fewer neighbours than common SNPs), and we have added an additional subsampling step to ensure the MAF distribution matches between the full dataset and the subset of SNPs used to fit the KDE. This approach is now implemented in the asthma applications and is further explained on line 383:

"We down-sampled the independent subset of SNPs to match the MAF distribution in this subset to that in the whole set of SNPs. This accounts for the confounding of LDAK weight and GWAS p-values by MAF: less common SNPs (MAF < 0.05) are over-represented among the independent subset and have, on average, larger p-values. Matching in this way prevents a bias of the KDE fit towards the behaviour of rarer SNPs."

The presentation of the power (sensitivity) from the simulations results by including any SNPs with r-squared above 0.8 with a causal variant may be overstating the power. More extensive studies are necessary to understand the impact of LD on both the power to detect causal SNPs and ability to control type 1 errors.

The reviewer raises a concern about the presentation of the power in our manuscript. If a SNP has r^2>0.8 with a causal variant then its expected marginal effect will not be 0, and thus must be non-null by definition. This is because we are aiming to find associated rather than causal SNPs, as is typical in GWAS (which can then be fine-mapped to find the causal SNPs if required).

To reassure the reviewer, we have explored how estimated sensitivity and specificity depend on the threshold values used to define ``truly associated SNPs'' and ``truly not-associated SNPs'' in our simulation analysis (Fig S8, Fig S9). Sensitivity increases slightly as the r2 threshold increases, as would be expected because the marginal effect at a SNP in LD with a causal variant increases as LD increases. Thus, our estimates of sensitivity in the main text are conservative, and likely to understate the sensitivity.

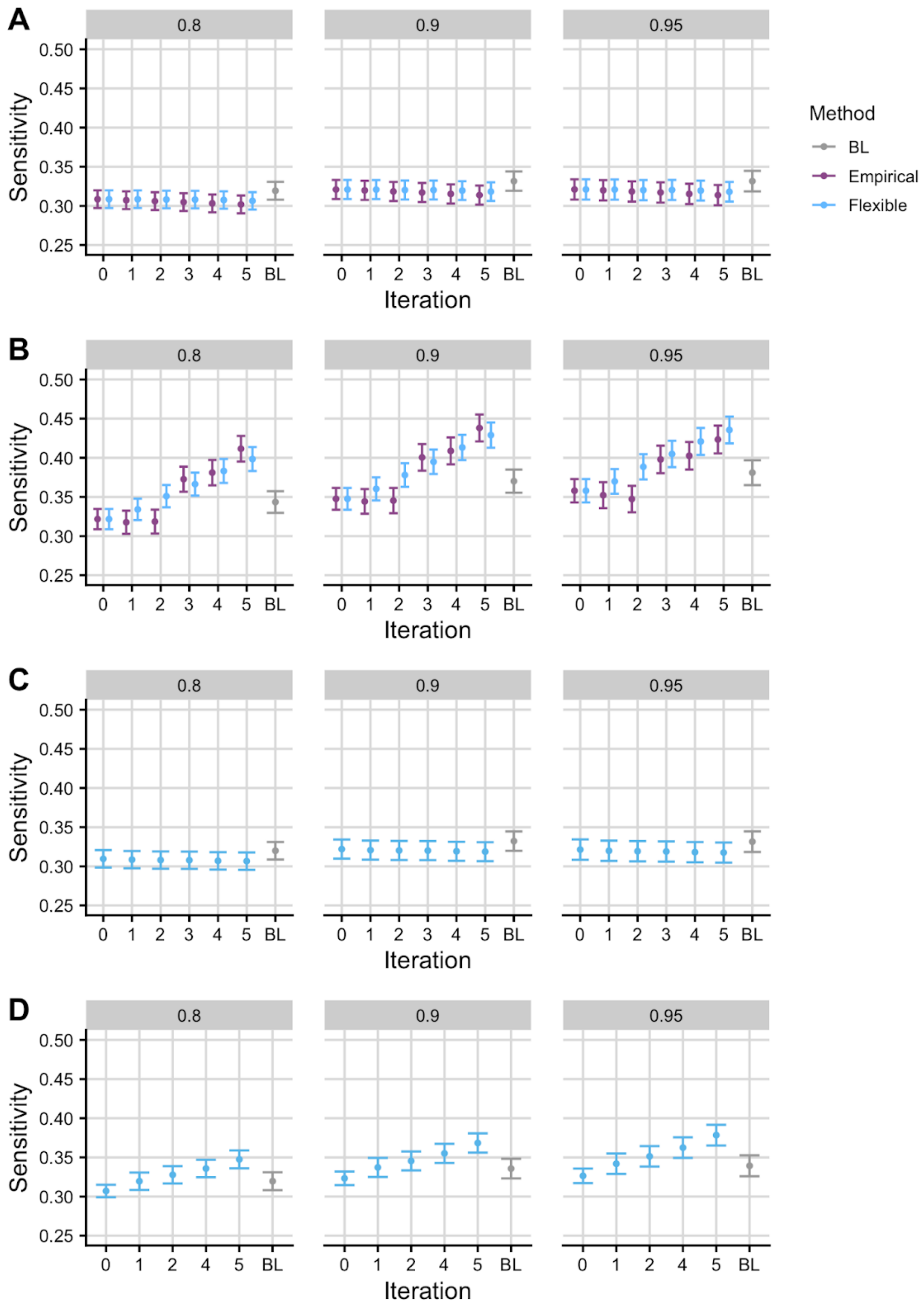

Fig S8 caption:
Simulation results assessing the sensitivity when increasing the r^2 value used to call associated SNPs. Mean +/- standard error for the sensitivity of FDR values from empirical and Flexible cFDR when iterating over independent (A; ``simulation A") and dependent (B; ``simulation B") auxiliary

data that is bounded by [0,1]. Panels C and D show the results from Flexible cFDR when iterating over independent (C; ``simulation C") and dependent (D; ``simulation D") auxiliary data simulated from bimodal mixture normal distributions. BL refers to results when using Boca and Leek's FDR regression to leverage the 5-dimensional covariate data. Iteration 0 corresponds to the original FDR values. Our sensitivity proxy is calculated as the proportion of SNPs with r^2\geq X with a causal variant (``truly associated"), that were detected with a FDR value less than 5e-06, where results are faceted for X=0.8, 0.9, 0.95. Results were averaged across 100 simulations.

With regard to the impact of LD on the ability to control type 1 errors, the specificity (1-FPR) decreases as the r2 value threshold used to define ``truly not-associated SNPs” increases, as expected since the marginal effect at a SNP in LD with a causal variant increases as LD increases.

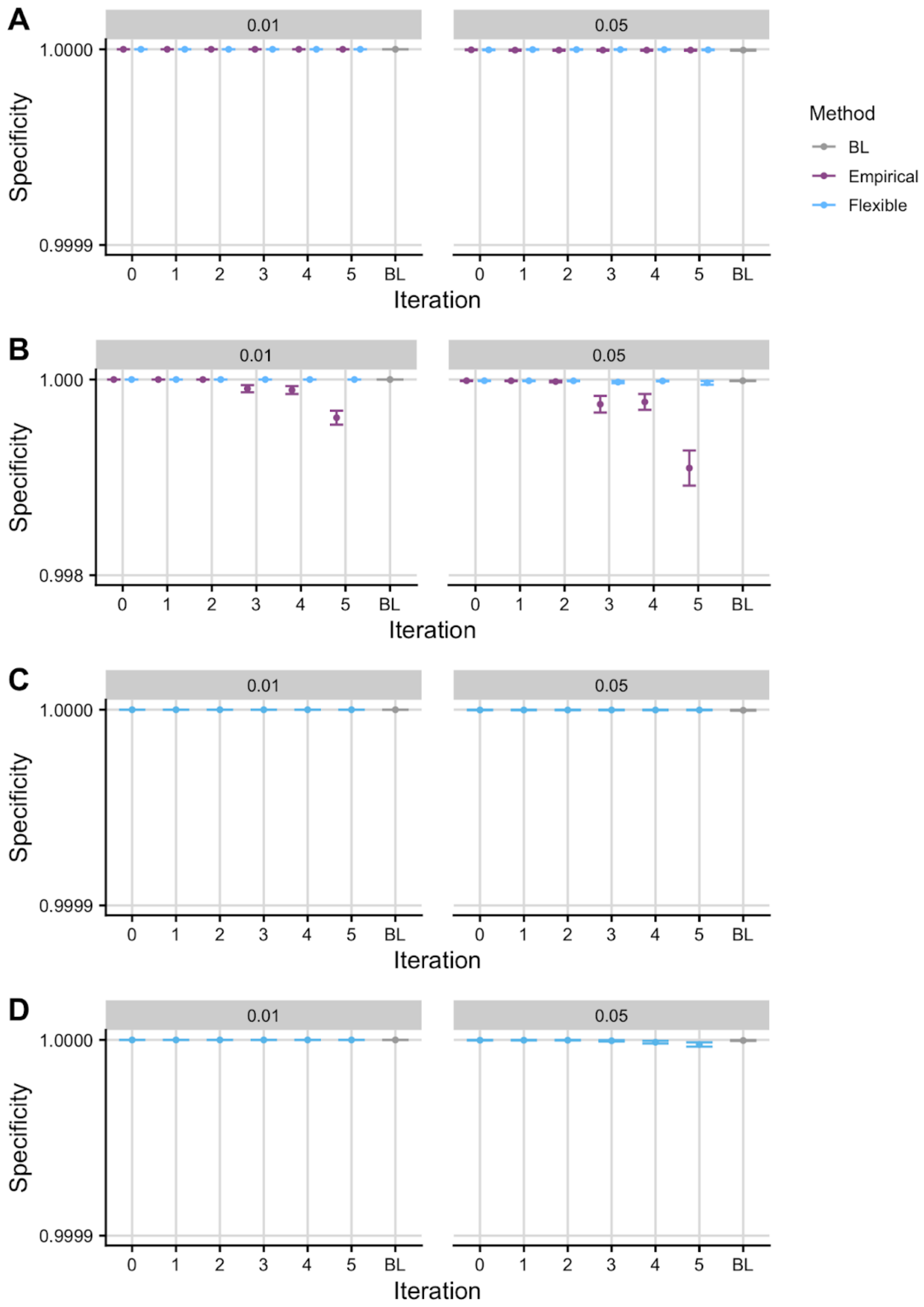

Fig S9 caption:
Simulation results assessing the specificity when increasing the r^2 value used to call not-associated SNPs. Mean +/- standard error for the specificity of FDR values from empirical and Flexible cFDR when iterating over independent (A; ``simulation A") and dependent (B; ``simulation B") auxiliary

data that is bounded by [0,1]. Panels C and D show the results from Flexible cFDR when iterating over independent (C; ``simulation C") and dependent (D; ``simulation D") auxiliary data simulated from bimodal mixture normal distributions. BL refers to results when using Boca and Leek's FDR regression to leverage the 5-dimensional covariate data. Iteration 0 corresponds to the original FDR values. Our specificity proxy is calculated as the proportion of SNPs with r^2\leq X with all the causal variants (``truly not-associated"), that were not detected with a FDR value less than 5e-06, where results are faceted for X=0.01, 0.05. Results were averaged across 100 simulations.

Minor Concerns:

To avoid confusion, it would be beneficial for the readers if the authors clarified how their definition of FDR (Equation 1) differs from the more commonly used form by Benjamini-Hochberg.

We agree with the reviewer that this clarification would be valuable to our manuscript and have included the following text in our new submission:

Line 109: *"This Bayesian definition of a tail area FDR \cite{efron2007} is asymptotically equivalent \cite{wen2018} to the FDR introduced by Benjamini and Hochberg \cite{benjamini1995}, which is the expected fraction of false discoveries amongst all discoveries."*

On page 4 (and throughout the paper) the authors refer to "controlling specificity". The word "control" is typically used in the context of limiting a quantity. It's more appropriate to say in this context that they wish to control the type 1 error rate or increase specificity.

We agree with the reviewer that this terminology was not appropriate and have amended the manuscript to read "increase the specificity" where appropriate.

Section 3.5.3 belongs in the supplement.

This section described how both empirical cFDR and Flexible cFDR were implemented in the simulation analysis. We have now additionally included details of how Boca and Leek's FDR regression was implemented in the simulation analysis. We feel that this section belongs in the main manuscript since it describes the key software functions and parameter values we used to implement these methods. We also do not have any supplemental text and it would be unusual to have a supplemental text consisting of a single short paragraph. That being said, if the reviewers or editors would prefer that this section was moved to the supplement, then we are open to making this change.

References

Liley, J., & Wallace, C. (2020). Accurate error control in high dimensional association testing using conditional false discovery rates. bioRxiv, 414318.
Korthauer, K., Kimes, P. K., Duvallet, C., Reyes, A., Subramanian, A., Teng, M., ... & Hicks, S. C. (2019). A practical guide to methods controlling false discoveries in computational biology. Genome biology, 20(1), 1-21.
Lei, L., & Fithian, W. (2018). AdaPT: an interactive procedure for multiple testing with side information. Journal of the Royal Statistical Society.

Yurko, R., G'Sell, M., Roeder, K., & Devlin, B. (2020). A selective inference approach for false discovery rate control using multiomics covariates yields insights into disease risk. Proceedings of the National Academy of Sciences, 117(26), 15028-15035.

Reviewer #3

In this article, the authors leverage the weighted false discovery framework popularized by Genovese, Wasserman and Roeder to look at two-dimensional extensions the authors term `conditional false discovery rate'. They present a derivation of a posterior probability conditionall on two statistics being sufficiently small. I have the following comments.

I have concerns about the lack of comparisons in the literature. Ploner et al. (2005, Bioinformatics) proposed a two-dimensional local false discovery rate that resolves many of the problems the authors describe in their `Conditional false discovery rate', `Flexible cFDR', and `Mapping p-value-covariate pairs to v-values' sections. This method is never discussed or compared with in the paper.

This paper certainly has very relevant work. In particular, the 2D fdr faces similar challenges about sparsity of data. They adapt the smoothing estimator proposed by Efron for the 1D fdr evaluating at discrete points; the authors suggest a 20x20 grid. However, the context of microarray and GWAS are very different. In microarray datasets, the number of variables is typically < 20,000 and correlation between genes is generally low to modest. In contrast, GWAS datasets contain upwards of 0.5 million variables, and correlation between SNPs in GWAS datasets is often substantial. The smoothed 2D fdr estimate is defined as the value which minimizes a penalized likelihood but calculating that (log) likelihood by summing log likelihoods over dependent observations would be invalid in the GWAS case due to LD. The importance of LD and the availability of many more data points mean we estimate the 2D KDE from an independent subset of SNPs, and derive the quantities in the cFDR estimator from that estimated KDE. However, the key difference in our view relates to the definition of local fdr as the ratio of probability density functions, compared to the FDR as the ratio of cumulative distribution functions, meaning that the latter is much less sensitive to sparsity of data away from the lower limits of its support. We now contrast these approaches in the discussion:

Line 886: *"Estimation of both FDR and local false discovery rates (often denoted ``fdr'') \cite{efron2004} require estimation of a ratio. In the case of FDR these are cumulative distribution functions (CDF), and in the case of the fdr these are probability density functions (PDF). The simple approach is to take a ratio of two separate estimates of the numerator and denominator. In the case of a 2d fdr proposed by Ploner et al. \cite{ploner2006}, this approach was found to be numerically unstable, because the sparsity of the data across 2 dimensions means the uncertainty attached to the estimate of the denominator in particular may be large. Ploner et al. \cite{ploner2006} proposed a solution based on binomial regression, adapting the Poisson regression method used by Efron \cite{efron2004} to estimate the denominator in the fdr ratio. Despite depending on a 2 dimensional density, we do not use a smoothing estimator for cFDR, yet it still performs well. This is because CDF estimates are typically more stable than PDF estimates at any given point (apart from towards the lower limits of the data), and is one of the attractive ideas for using cumulative rather than probability density functions."*

We also reference Ploner's method within the bracket of related statistical methods in the re-written Introduction section.

Upon further reading, there is no comparison done with any other methods, which again is a limitation of the paper.

In our original submission we included a comparison with FINDOR and GenoWAP. In our new submission we additionally compare Flexible cFDR to Boca and Leek's FDR regression and IHW. We find that Flexible cFDR is generally superior in terms of sensitivity, specificity and versatility.

Brad Efron typically transforms the p-values back to a normal distribution scale. Given the authors' assumptions in equation (2), couldn't a conceptually simpler approach be

Inverse transform the p's and q's separately using the normal cdf.

Fit a two-component mixture model with bivariate normal distributions and use the posterior probabilities to derive local false discovery rates.

This is an attractive approach, and similar to the approach taken in https://www.nature.com/articles/ng.3751. However, a key extension in this work is to encompass q which may not be some transform of a mixture Gaussian distribution, which is why we chose a KDE over a specified form of parametric model. We have re-written the Methods section to better describe how Flexible cFDR removes any parametric assumptions placed on the auxiliary data.

The authors are working in the auxiliary information framework for multiple testing, but it might be use to describe what are examples of q_i sooner than the simulations and results sections.

We agree with the reviewer that it is very important to highlight specific use-case examples early on in a manuscript and regret that this was not done in our initial submission. In our new submission, we state the types of auxiliary data that can be leveraged in the Abstract, Author Summary and the Introduction. The Flexible cFDR web page also includes a vignette describing the type of auxiliary data that can be leveraged and provides links to these data sources (https://annahutch.github.io/fcfdr/articles/extra-information.html).

I found several of the statements the authors make in the paper to be incorrect or confusing.

Line 87ff: I don't understand why you want to use conditional here when the main extension/innovation would be conditioning in two-dimensional space. The early literature on Empirical Bayes and false discovery rates talked about P(null|p-value <= a), which would also be a conditional quantity.

We thank the reviewer for recognising this potentially confusing use of terminology. However, this is not our terminology but rather that introduced by Andreassen and colleagues in their initial cFDR paper from 2013 in PLOS Genetics, and is now used throughout the cFDR literature. We did not want to introduce new terminology into the cFDR literature, as this may serve to confuse readers.

That being said, we recognise that it may be confusing to readers that "conditional" FDR is called so even though the original FDR also conditions on a quantity. We have now clarified this in the manuscript and we thank the reviewer for pointing out this potential ambiguity in the naming convention which may confuse readers if not clarified properly:

Line 112: *"Given additional p-values, q_1,...,q_m, for the same m SNPs for a ``conditional trait'', the Bayesian FDR can be extended to the conditional Bayesian FDR (cFDR) by conditioning on both the*

principal and the conditional trait variables (in contrast to the standard FDR which conditions only on the principal trait variable)."

Lines 105 – 109: I don't get the transition between the two sentences.

We agree with the reviewer that this could have been phrased better. We meant that the standard cFDR derivation holds for continuous q, but that the current method used to approximate the cFDR may not be accurate for continuous q since they use empirical CDFs which can be inaccurate in regions of sparse data (which is more common for arbitrary q). We wanted to develop an extension of the cFDR estimator which would be robust in these sparse data regions. We have re-structured and re-written the Methods section so that our motivations for developing Flexible cFDR are made more clear. Specifically, we have re-written the paragraph the reviewer refers to:

Line 149: *"The cFDR estimator in Eq 5 holds in the more general setting where q_1,...,q_m are real continuous values from some arbitrary distribution that is positively stochastically monotonic in p. However, the current methods to estimate the cFDR use empirical CDF estimates which can be inaccurate in regions of sparse data. These sparse data regions are likely to be found more often in unbounded auxiliary data from arbitrary distributions (for example near the extreme observations) than auxiliary data that are p-values from related traits, and thus bounded by [0,1]. Moreover, the method used to control the frequentist FDR \cite{liley2021} requires normally distributed auxiliary data. We describe a new, more versatile cFDR framework for data pairs consisting of p-values for the principal trait (p) and continuous covariates from more general distributions (q). We call our method ``Flexible cFDR''."*

Line 172: how do you justify 10% as opposed to another percentage?

We extend the range of the data by 10% on either side to fit the KDE to ensure that the integral of the KDE approximated in our method equals 1 (i.e. we haven't inadvertently trimmed some of the underlying distribution). This is an arbitrary value, and one that we chose on the basis of visualising the data and the fitted density. In our experience, we did not encounter any instances where 10% was problematic.

To reassure the reviewer, in our updated accompanying software we now include plots to allow users to visualise the fit of the KDE to their data, they can then adjust this percentage if required.

Line 232ff: I really don't understand the comments about "iterative" usage. Usually, we have one dataset, run the FDR analysis and determine the genes. That is not iterative.

From the reviewers comment it is clear that in our initial submission we did not effectively explain the purpose of iteration in Flexible cFDR. The iterations are used to leverage various types of auxiliary data in turn for the same principal trait p-values, and we believe are a key selling point of our method. This is analogous to the relevant statistical methods which permit multiple covariates. The ChIP-seq application in the manuscript also illustrates the iterative usage in practise (leveraging H3K27ac fold change values in different asthma relevant cell types). We have now clarified this in the manuscript:

Line 225: *"The derivation of v-values also allow for iterative usage, whereby the v-values from the previous iteration are used as the ``principal trait'' p-values in the current iteration \cite{liley2021},*

thus allowing users to incorporate additional layers of auxiliary data into the analysis at each iteration, akin to leveraging multi-dimensional covariates."

Results: I found it very hard to see how the proposed method was gaining/doing better relative to comparators.

In our new submission, we now compare results from Flexible cFDR to those from four comparator methods and have re-written our Results section to better highlight the relative gains of our method. We have also updated our simulation-based analysis to show that Flexible cFDR maintains FDR control whilst certain comparator methods do not.

Using a subscript on the null hypothesis of P when the p-values are denoted by p_1,…,p_n is confusing

We are unsure of what the reviewer is referring to here. If they mean the *superscript* used in the notation of the null hypothesis, then this is the notation used in the existing cFDR literature, such as Liley and Wallace (2021; Biometrical Journal). We did not want to introduce new notation as this may serve to confuse readers.
