# Supplementary material for: Leveraging auxiliary data from arbitrary distributions to boost GWAS discovery with Flexible cFDR
Source: PLoS Genet. 2021 Oct 20;17(10):e1009853. doi: 10.1371/journal.pgen.1009853 (PMC8559959; doi:10.1371/journal.pgen.1009853)
Supplement: Attachment — Submitted filename: review-response.pdf [file pgen.1009853.s022.pdf]

__**Response to reviewers comments**__

Reviewer #1: The authors have done a good job of addressing comments from the previous round of reviews.

Reviewer #2: The manuscript, "Leveraging auxiliary data from arbitrary distributions to boost GWAS discovery with Flexible cFDR", describes an FDR approach for GWAS in the presence of secondary data-sets such as GWAS summary statistics of related traits or variant-level annotation scores. The methods is based on a widely accepted concept, the conditional false discovery rate (cFDR). It overcomes a major limition of previous work that the secondary data-set must follow a mixture Gaussian distribution, thus enabling the application of the proposed approach to integrating a variety of secondary data-sets for GWAS on a complex trait, in particular functional genomic features. The data application is thorough, with examples from large GWAS (UK Biobank) integrating predicted variant function scores (GenoCanyon) and epigenomic features (H3K27ac marks).

Overall I find the manuscript well-written. The method, albeit a seemingly straightforward extension to a recent work by the same research group, is a timely and highly useful contribution to the current "multi-omics" study theme for many complex trait studies. It could be a good addition to the omics data integration toolbox. However, I think the manuscript can benefit a lot with improved Methods section, to emphasize the novelty and contrast more with the published empirical cFDR method.

1. In Introduction I suggest adding some narrative to motivate the method with biological applications. Current manuscript simply mentions in passing that the approach can integration functional genomic data. Even for GWAS data integration, would the new method help to integrate Bayes Factors without having to convert them to z-scores? A list of concrete example along with a few citations of existing approaches to integrate such data with GWAS will help the readers appreciate the contribution of the new method.

We thank the reviewer for their comment and we agree that some additional narrative to motivate the method with a biological application would help readers to appreciate the contribution of Flexible cFDR to the field. The reviewer is correct, in that other auxiliary measures from GWAS could also be leveraged by Flexible cFDR, including Bayes factors. In our new submission we have added an additional paragraph to the Introduction based on these suggestions:

Line 93: *“Integrating functional genomic data with GWAS test statistics is not a new concept, and is motivated by SNP enrichment studies \cite{maurano2012, schork2013, cano-gamez2020, pickrell2014}. For example, Pickrell \cite{pickrell2014} found that loci that associated with serum high-density lipoprotein (HDL) concentrations were enriched for several functional annotations, including enhancer regions in HepG2 cells and coding exons. When using their ``fgwas” model to integrate these functional annotations with GWAS test statistics, they were able to identify new loci that robustly associated with HDL concentrations. However, the fgwas approach outputs posterior probabilities rather than p-values, as are typical in GWAS, and currently only supports binary auxiliary data. In contrast, Flexible cFDR outputs quantities analogous to p-values and also supports a wide range of auxiliary data types, including (but not limited to) continuous data derived from*

functional genomic experiments (e.g. fold change values from ATAC-seq or ChIP-seq) and GWAS-related values (e.g. allele frequencies, sample sizes, Bayes factors or p-values).”

In the Introduction of our original submission we described two additional approaches (FINDOR and GenoWAP) that integrate functional annotations with GWAS test statistics, but these were not introduced in a biological context. In our new submission, we have rewritten the relevant sentences to better emphasis the specific biological applications of these approaches:

Line 42: “In the GWAS setting, the FINDOR method (which was shown to be generally less powerful, but superior in terms of false positive findings, to stratified FDR, GBH and IHW methods) was developed to leverage auxiliary data relating to how well SNPs tag functional categories that are enriched for heritability with GWAS test statistics \cite{finucane2015, gazal2017, kichaev2019}.”

Line 65: *“GenoWAP was developed to leverage scores of SNP functionality (called ``GenoCanyon scores'' \cite{lu2015}) with GWAS test statistics and includes a thresholding step to define ``functional'' SNPs.”*

2. In Methods, please explicitly summarize key differences between Flexible cFDR and empirical cFDR, and why this is relevant to the proposed application -- it helps to simply listing examples of functional annotation scores that are obviously not a mixture of Gaussian. Also please explain what it is meant by "sparse data" and how exactly it may impact the cFDR approach because I can think of versions of Gaussian mixture that handles sparse data; unless I'm wrong about what sparse data means in this context.

We agree that it is important to summarise the key differences between Flexible cFDR and Empirical cFDR, and we have rewritten the paragraph beginning at line 166 to do this.

This paragraph also relates to ``sparse data’’ which was not fully explained in our original submission. [The sparse data that we refer to relates to the standard definition (gaps between data points) and the inaccuracy of empirical CDFs for sparse data (empirical CDFs are step functions between data points, and so may be inaccurate between data points). This corresponds to the estimation of the cFDR and does not correspond to the Gaussian mixture that is assumed when deriving v-values later on in the method.]

Line 166: *“The cFDR estimator in Eq \eqref{cfdr_est} holds in the more general setting where $q_1,...,q_m$ are real continuous values from some arbitrary distribution that is positively stochastically monotonic in p. However, the current methods were designed with specific assumptions about the distribution of the auxiliary data (p-values from related traits and thus bounded by $[0,1]$). Sparse data regions are likely to be found more often in unbounded auxiliary data from arbitrary distributions (for example near the extreme observations) and empirical CDFs are typically inaccurate in sparse data regions because they are step functions. Moreover, the method used to control the frequentist FDR \cite{liley2021} assumes auxiliary data can be modelled using a mixture of centered Gaussian distributions, meaning that it is not yet applicable for auxiliary data from arbitrary distributions. We consequently describe a new, more versatile cFDR framework for data pairs consisting of p-values for the principal trait (p) and continuous covariates from*

more general distributions (q). We call our method ``Flexible cFDR'' and show that it is naturally suited to leveraging functional genomic data, which is not typically Gaussian."

Please also note that in the Discussion, once all Methods and Results have been presented, we explicitly list the key differences between Flexible cFDR and Empirical cFDR (we have updated this narrative very slightly since our initial submission, to make it clearer):

Line 900: *"Our manuscript describes four key advances enabling the extension of the cFDR framework to the functional genomics setting. Firstly, we derive an estimator based on a 2-dimensional KDE of the bivariate distribution rather than empirical estimates, making our method considerably faster than earlier empirical approaches. Secondly, the cFDR framework estimates $q|H_0^p$ with the relatively coarse approximation $q|p > 1/2$. In contrast, Flexible cFDR utilises the local false discovery rate in its estimator for $q|H_0^p$. The local false discovery rate conditions the probability of a null hypothesis on the point value of its p-value, and its use allows for finer-grained estimation of $q|H_0^p$. Thirdly, we remove the assumption that $q|H_0^p$ can be transformed to a mixture of centered normals, and instead integrate over the previously estimated KDE, which relaxes the distributional assumptions placed on the auxiliary data. Finally, Flexible cFDR is supported by user-oriented software documented on an easy-to-navigate website (\url{https://annahutch.github.io/fcfdr/}). The website features several fully reproducible vignettes which illustrate how the method can be applied to a particular data set at the desired level of error control. We hope this support will make Flexible cFDR accessible to a wider range of researchers."*

We understand the importance of highlighting the specific biological applications of Flexible cFDR and have updated the Methods section to direct readers to the software website, which describes an application that leverages a wide variety of genetic and genomic data (https://annahutch.github.io/fcfdr/articles/t1d_app.html) and also a list (including links) of functional genomic data that can be leveraged (https://annahutch.github.io/fcfdr/articles/extra-information.html).

Line 276: *"Flexible cFDR supports a wide range of auxiliary data types and is particularly suited to leveraging functional genomic data, which is not typically Gaussian (e.g. fold change values from ChIP-seq or per-SNP scores of functionality). We include vignettes (\url{https://annahutch.github.io/fcfdr/articles/extra-information.html}; \url{https://annahutch.github.io/fcfdr/articles/t1d_app.html}) exemplifying the types of functional genomic data that can be leveraged and also describing how the LDAK method \cite{speed2020} can be used to generate an independent subset of SNPs for input into the software."*

3. One improvement is the use of a flexible threshold rather than a hard threshold of 1/2 when computing cFDR. I can think of a specific version of cFDR without using such a threshold as described in the manuscript, but still uses mixture Gaussian for the auxiliary data. Is that preferable for when the secondary data is also GWAS results, or the new flexible cFDR should replace the empirical cFDR approach for all applications?

As the reviewer suggests, one can imagine a method that uses flexible thresholding to approximate Q|H0 and also the mixture Gaussian approach to derive v-values. We believe that Empirical cFDR could potentially be improved using this flexible thresholding approach,

but that this is outside the scope of the current manuscript. Flexible cFDR was explicitly designed for auxiliary data that requires alternative modelling approaches (than mixture Gaussians) whereas Empicial cFDR was explicitly designed for auxiliary data that can be transformed to mixture Gaussians. Therefore, Empirical cFDR may be preferable for GWAS data and we agree that it would be interesting to explore an extension of Empirical cFDR that uses flexible thresholding.

4. A concern on line 169 is that it uses a Gaussian kernel. While this is a common KDE choice, is it good enough for arbitrary distribution of secondary data (which can be a positive score, a probability, a binary variable or on phred scale)? Please justify.

There are numerous forms of auxiliary data that can be leveraged by Flexible cFDR (any auxiliary data that is continuous and positively stochastically monotonic in p) and it would be impossible to explore how the Gaussian kernel performs on all possible auxiliary data types. Instead, our accompanying software outputs a plot (by default) to allow users to visualise the fit of the KDE to their data. In our experience, we did not encounter any instances where the Gaussian kernel was problematic, but we encourage users to scrutinise the fit of the KDE to their data.

In our original submission we discussed how the scale that the auxiliary data is measured on may impact the KDE. In our new submission, we have re-written this paragraph to discuss in more detail the KDE fitting procedure and the use of a Gaussian kernel.

Line 915: *"One can see that the performance of Flexible cFDR depends on how well the KDE fits the data. Usual concerns about KDE apply, including that fits may be poor if there are regions with very sparse data. The auxiliary data can be transformed to improve the KDE fitting procedure, as in application 2 where we log-transformed the raw fold change values to avoid long tails. The optimal scale for the auxiliary data is likely to depend on the relationship between the principal p-values and the auxiliary data, and is not something we have explored here, but as usual, data visualisation is likely to be helpful to confirm that the scale for the auxiliary data is sensible. By default, the Flexible cFDR software returns a plot of the fitted 2D KDE and the estimated density of the auxiliary data overlaid onto the real data values, enabling users to visually examine the fit of the KDE to their data. Investigating the KDE fitting procedure, including the suitability of the Gaussian kernel, is an interesting area for future research."*

We note that the reviewer suggests using Flexible cFDR on binary or discrete data. Flexible cFDR requires *continuous* auxiliary data, which we did not emphasise sufficiently in our original submission. In our new submission, we have added a paragraph to the Discussion describing how Flexible cFDR requires continuous auxiliary data, but could potentially be extended to support discrete or binary data in the future.

Line 954: *"Secondly, Flexible cFDR requires that the auxiliary data to be leveraged is continuous. This means that the approach cannot currently be used to leverage functional genomic data that yields discrete or binary values, such as PHRED scores, whether SNPs are synonymous or non-synonymous or whether they reside in coding regions of the genome. A fruitful contribution to the field would be to extend the cFDR approach to support discrete or binary data, thus increasing applicability."*

Also some additional high level comments:

1. Alternative to cFDR, various multivariate analysis approaches (eg bi-variate GWAS) and data integration methods (eg fGWAS or those based on stratified LDSC) can perform data integration. These are completely different, but possibly more popular alternatives, to cFDR. Please discuss cFDR in the context of these methods.

There are many methods that have been developed for covariate-based multiple hypothesis testing in the context of GWAS. In our manuscript, we chose to compare our approach to existing methods which have previously been shown to outperform other approaches (e.g. see https://genomebiology.biomedcentral.com/track/pdf/10.1186/s13059-019-1716-1.pdf). We did not include comparisons with multivariate approaches, such as bivariate GWAS, as we have only seen these presented in the context of association statistics, not functional genomic data.

fgwas is a hierarchical Bayesian approach that re-weights genomic blocks based on functional data. We did not include a direct comparison with fgwas in our manuscript because the fgwas software does not currently support continuous annotations, so could not be used to leverage the same auxiliary data as that used in our simulations or applications. We agree that fgwas is an important approach and now describe details of how it differs to Flexible cFDR in our new submission:

Line 94: "*For example, Pickrell \cite{pickrell2014} found that loci that associated with serum high-density lipoprotein (HDL) concentrations were enriched for several functional annotations, including enhancer regions in HepG2 cells and coding exons. When using their ``fgwas" model to integrate these functional annotations with GWAS test statistics, they were able to identify new loci that robustly associated with HDL concentrations. However, the fgwas approach outputs posterior probabilities rather than p-values, as are typical in GWAS, and currently only supports binary auxiliary data. Instead, Flexible cFDR output quantities analogous to p-values and also supports a wide range of auxiliary data types, including (but not limited to) continuous data derived from functional genomic experiments (e.g. fold change values from ATAC-seq or ChIP-seq) and GWAS-related values (e.g. allele frequencies, sample sizes, Bayes factors or p-values).*"

The FINDOR method, which we directly compared to Flexible cFDR in our manuscript, is based on stratified LDSC. We found that FINDOR had greater sensitivity than Flexible cFDR and hypothesised that this was due to the many more annotations that were leveraged by the approach (e.g. because the baseline-LD model v2.2 contains 96 annotations). A major advantage of Flexible cFDR compared to FINDOR (and likely other methods based on stratified LDSC) is the accessibility of the method. We struggled ourselves when implementing FINDOR due to the two-step approach that is required (running stratified LDSC then FINDOR). In contrast, users are able to run Flexible cFDR using one line of R code and the accompanying web-page guides users through several fully reproducible vignettes to enhance accessibility of the approach.

2. Related to comment above, one limitation of cFDR is that it seems to allow for one auxiliary data-set at a time. Please comment on applications where multiple functional annotations need to be integrated. The application of H3K27ac data integration seems to simply average over different tissues and cell types?

A selling point of the cFDR approach is that it can be applied iteratively to incorporate multiple auxiliary data sets. Based on the reviewers comment this is not something that we described effectively in our original submission. We have rewritten the following sentence to better describe this feature:

Line 248: *“The derivation of v-values also allow for iterative usage, whereby the v-values from the previous iteration are used as the ``principal trait'' p-values in the current iteration \cite{liley2021}. This allows users to incorporate additional layers of auxiliary data into the analysis at each iteration, akin to leveraging multi-dimensional covariates, and this approach is exemplified both in our simulation analysis and in our application utilising ChIP-seq data.”*

However, it is important that each iteration considers new information, and does not iterate over layers containing essentially the same information. The H3K27ac data was averaged because it clearly fell into two clusters (Fig. S6). We averaged the data for cell types that clustered together and then applied cFDR iteratively. We have now better clarified this in the Discussion.

Line 941: *“Our method has several limitations. Firstly, if applying Flexible cFDR iteratively then it is important that each iteration considers new information. That is, care must be taken to ensure that the auxiliary data to be leveraged iteratively is capturing distinct disease-relevant features to prevent multiple adjustment using the same auxiliary data.”*

Minor points on the methods:

1. Please briefly recap the definition of boundary effect in KDE.

We have now re-written the corresponding paragraph and added a citation to Silverman's ‘Density Estimation for Statistics and Data Analysis’ book which describes the boundary effect and the ‘reflection technique’ which we use.

Line 185: *“Since the absolute Z-scores are bounded by 0, the KDE will penalise the lack of negative data points and may underestimate the true density in regions close to 0. To avoid this boundary effect, we mirror the absolute Z-scores onto the negative real line together with their associated Q values but only estimate the KDE on the non-negative part of the data, akin to the ``reflection technique'' described by Silverman \cite{silverman1986}.”*

2. Line 214 please refer to eqn. (9) to clarify how it works.

We have updated the relevant paragraphs to better illustrate how this step works.

We therefore have that

$$\widehat{Pr(Q=q|H_0^p)} = \frac{\widehat{Pr(Q=q,H_0^p)}}{\widehat{Pr(H_0^p)}}, \quad (9)$$

where $\widehat{Pr(Q=q,H_0^p)}$ is derived by integrating Eq 8 over P and $\widehat{Pr(H_0^p)}$ is derived by integrating Eq 8 over P and Q. We then integrate over Q to obtain

$$\widehat{Pr(Q\leq q|H_0^p)} = \frac{\widehat{Pr(Q\leq q,H_0^p)}}{\widehat{Pr(H_0^p)}}. \quad (10)$$

where we use $\hat{}$ to denote that these are estimates under the assumption $H_0^p \perp\!\!\!\perp Q|P$.

Our final cFDR estimator is therefore:

$$\widehat{cFDR}(p,q) = \frac{p\times \widehat{Pr(Q\leq q|H_0^p)}}{\int_{-\infty}^{q}\int_{z_p}^{\infty} f(x,y)dxdy}. \quad (11)$$

where z_p is the Z-score associated with p.

And also:

conditional on H_0^p is the standard uniform density). We estimate $f_0^q(q)$ as an intermediate step in the derivation of $\widehat{Pr(Q\leq q|H_0^p)}$ (Eq 9).

Reviewer #3: The authors have proposed a new method, flexible cFDR, that generalizes the conditional FDR approach to incorporate auxiliary data information in GWAS of a trait. Usually the auxiliary data incorporated in existing method include p-values from a related trait. Here the authors have leveraged auxiliary data from arbitrary distributions such as functional genomic data. They have done extensive simulation analysis and benchmarking. They also performed two real data analyses with validation to illustrate properties of their approach against existing ones. In my opinion, the authors have done a good job of addressing previous reviewer comments. I have no additional comments; just the 3 very minor comments below.

We thank the reviewer for their comments and have addressed their minor comments below.

Minor Comments

1. If possible, provide links from which GenoCanyon scores and ChIP-seq counts were downloaded.

We have added these relevant links to the manuscript.

Line 436:

“We downloaded GenoCanyon scores from \url{http://zhaocenter.org/GenoCanyon_Downloads.html} for each of the 1,968,651 SNPs that GWAS p-values were available for, noting a bimodal distribution for the scores (S5A Fig).”

Line 446:

“We downloaded consolidated fold-enrichment ratios of H3K27ac ChIP-seq counts relative to expected background counts from NIH Roadmap \cite{bernstein2010} (\url{https://egg2.wustl.edu/roadmap/data/byFileType/signal/consolidated/macs2signal/foldChange/}) in primary tissues and cells relevant for asthma: immune cells and lung tissue.”

2. Suggest that scientific notation be used instead of E-notation for small numbers like p-values.

In our new submission, we have switched to scientific notation rather than E-notation throughout.

3. Currently, the article is a tad lengthy. Perhaps the authors can move some details in the Methods and the Results sections to the supplementary.

We feel that all sections are suitable for the main manuscript, but if the reviewer or editors feel strongly about the length of the manuscript then we are happy to make this change.
