# Supplementary material for: Leveraging auxiliary data from arbitrary distributions to boost GWAS discovery with Flexible cFDR
Source: PLoS Genet. 2021 Oct 20;17(10):e1009853. doi: 10.1371/journal.pgen.1009853 (PMC8559959; doi:10.1371/journal.pgen.1009853)
Supplement: Attachment — Submitted filename: Responses to comments.pdf [file pgen.1009853.s023.pdf]

Reviewer #2: The authors have satisfactorily addressed and clarified my previous concerns. My only remaining question on the method relates to the iterative approach to incorporate multiple auxiliary data. Different from a formal multivariate analysis, the order of data-sets entering the iterative approach seem to matter. If that is the case, is the result sensitive to the order of input? I suggest the authors show with theoretical or empirical results that this is not a concern, or acknowledge it as a short-coming for multiple auxiliary data.

Thank you for your comment. Our previously submitted manuscript stated:

Line 703: *"We also found that the order of which we iterated over the auxiliary data had minimal impact on the results (S17 Fig)."*

And included a supplemental figure (included below) showing that in our H3K27ac ChIP-seq application, Flexible cFDR was robust to the order of which the data-sets entered the iterative approach.

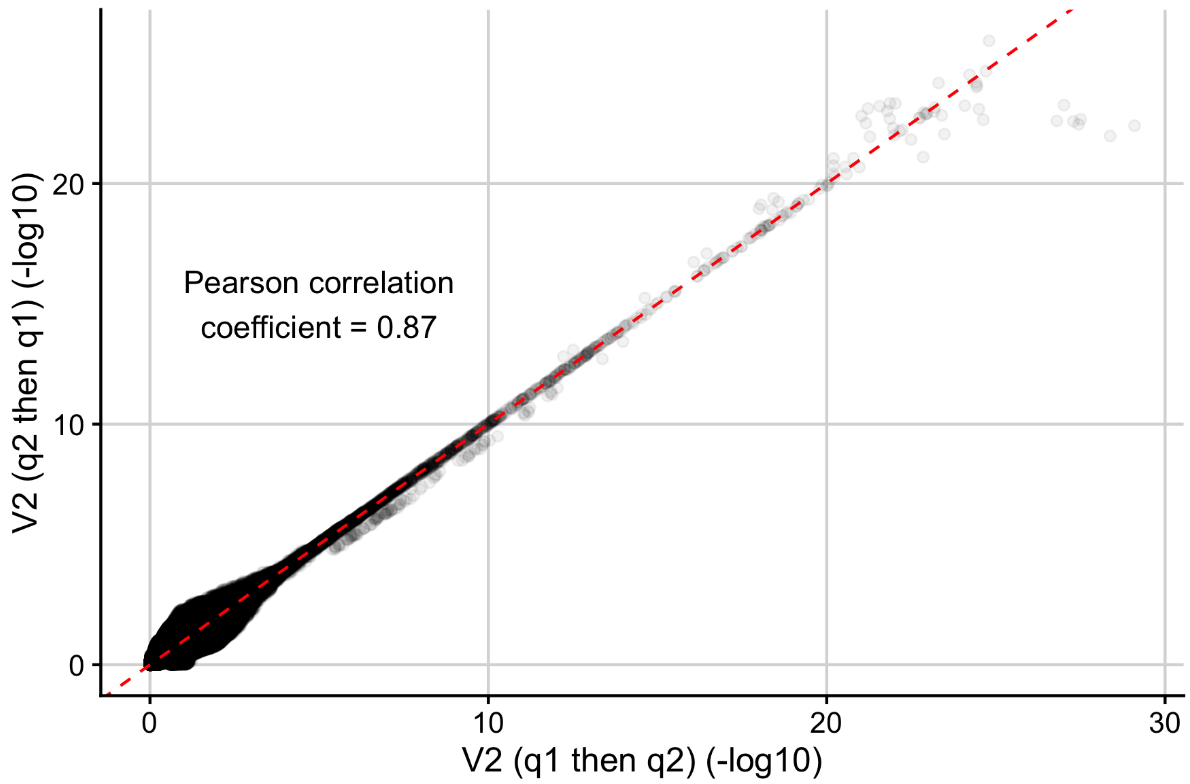

Caption: Switching the order of iteration. (-log10) v-values after 2 iterations of Flexible cFDR leveraging H3K27ac data when iterating over q2 and then q1 against (-log10) v-values when iterating over q1 then q2.

We regret that we did not make this clear enough in our previous submission. In our new submission we have re-written this sentence and have also added text to the discussion highlighting this point.

Line 703: *"One could expect that the order of which the auxiliary data is iterated over may impact the results from Flexible cFDR. Reassuringly, in this application we found that the order of which we iterated over the auxiliary data had minimal impact on the results (S17 Fig)."*

Line 876: *"A related extension would be to formally assess robustness of the cFDR approach to the order of which the auxiliary data is iterated over."*

Reviewer #3: I had only minor comments in the previous review, which the authors have addressed. I have no more comment to add.
